# Microscopic Leaching Mechanism of Fly Ash Geopolymer (FAG) Under Coupled Stray Current and Soft Water

**DOI:** 10.3390/polym18151883

**Published:** 2026-07-31

**Authors:** Fang Liu, Zhihao He, Ran Tang, Xinchao Zheng, Baomin Wang, Xiaojun Wang, Xiaosa Yuan

**Affiliations:** 1 Shaanxi Key Laboratory of Safety and Durability of Concrete Structures, Xijing University, Xi’an 710123, China; 2 College of Civil Engineering and Architecture, China Three Gorges University, Yichang 443002, China; 3 School of Civil Engineering, Dalian University of Technology, Dalian 116023, China

**Keywords:** fly ash geopolymer, stray current, soft water, coupled leaching, microscopic mechanism, pore structure

## Abstract

Severe electrochemical corrosion and leaching degradation of underground structures are induced by coupled stray current and groundwater in rail transit systems like subways. The microstructural evolution of fly ash geopolymer (FAG) during accelerated leaching was systematically investigated by simulating a coupled direct current (DC) stray current and soft water environment. Pore solution alkalinity and electrolytic cell OH^−^ concentration were utilized as evaluation indicators, combined with X-ray diffraction (XRD), Fourier transform infrared spectroscopy (FTIR), scanning electron microscopy (SEM), and mercury intrusion porosimetry (MIP). Results indicate that substantial OH^−^ leaching occurs under the coupled stray-current and soft-water exposure, leading to reduced pore-solution alkalinity and changes consistent with the degradation of the N–A–S–H binding network. Consequently, porosity, most probable pore diameter, and the quantity of harmful pores are increased. However, no obvious changes in the major diffraction peaks associated with quartz and mullite were detected within the resolution of the qualitative XRD analysis. Furthermore, a linearly decreasing trend over time is observed for the coupled leaching rate. Mix proportion analysis demonstrates that FAG leaching resistance is improved by reducing the water-to-binder ratio; specifically, superior gel phase content and pore structure are maintained at a ratio of 0.30. Additionally, cumulative OH^−^ leaching is effectively reduced by decreasing the sodium silicate modulus, with optimal resistance exhibited between 1.0 and 1.2. Concurrently, pore solution alkalinity before and after leaching is significantly elevated by increasing the alkali dosage. This exerts a pronounced inhibitory effect on OH^−^ leaching, thereby substantially enhancing the overall leaching resistance.

## 1. Introduction

New pathways for resolving urban traffic congestion during the urbanization process have been provided by the vigorous development of urban rail transit [1,2,3]. However, since direct current (DC) traction power supply systems are widely adopted in rail transit networks such as subways, complete insulation between the running rails and the track bed is difficult to achieve. Consequently, “stray current” leaking into structural components and the surrounding earth is inevitably generated [4]. Extremely severe electrochemical corrosion is induced by this stray current. Not only is the corrosion of underground metal pipelines and internal steel reinforcement within the concrete accelerated, but severe leaching degradation of the concrete matrix in groundwater (soft water) environments is also triggered, which poses a tremendous threat to the safety and durability of subway structures [5,6,7]. Therefore, advanced engineering materials possessing excellent corrosion resistance are urgently sought to resolve this critical issue in the field of rail transit construction.

Geopolymer, as a novel low-carbon inorganic cementitious material, is typically synthesized through the reaction of aluminosilicate-rich industrial solid wastes, such as fly ash and slag, activated by alkaline activators [8,9,10]. Due to its unique three-dimensional network-like amorphous or semi-crystalline microstructure (with N-A-S-H or C-A-S-H gels as the primary products), superior early strength, high-temperature resistance, and outstanding chemical attack resistance (e.g., against acid, sulfate, and chloride ions) are exhibited by geopolymers compared to traditional Portland cement [10,11,12]. Previous investigations of geopolymer durability have mainly focused on acid attack, sulfate and chloride exposure, carbonation, and water-induced leaching. Under acidic conditions, proton attack and alkali loss may promote the rupture of Si–O–Al and Si–O–Si bonds, accompanied by dealumination or dissolution of the aluminosilicate gel. Under sulfate- and chloride-containing environments, durability is closely associated with ion transport, chemical binding or precipitation, and pore-network connectivity. In aqueous leaching environments, the release of chemical components from monolithic geopolymers may be governed by diffusion, surface wash-off, solubility control, and progressive depletion. These studies demonstrate that geopolymer durability is jointly controlled by precursor chemistry, gel composition, pore structure, and the external exposure environment [13,14].

Although extensive research has been conducted by scholars worldwide regarding the deterioration effect of stray current on cementitious materials, existing achievements are primarily focused on traditional cement-based materials [15,16,17]. As early as 1997, accelerated leaching on cement-based materials was performed by the Japanese scholar Saito [18] using an applied electric field to investigate the deterioration effect of electric-field-accelerated leaching. It was discovered by Bertolini et al. [19] that the corrosion of steel reinforcement in concrete is accelerated by stray current, and it was pointed out that the stray current corrosion process is further accelerated by an increase in chloride concentration within the aggressive environment. The influence of an electric field on the thaumasite form of sulfate attack (TSA) in cement-based materials was investigated by Luo et al. [20] through a comparison between electric field acceleration and a full-immersion environment. It was revealed that the TSA mechanism is not altered by the electric field; rather, the attack process is accelerated by promoting the penetration of aggressive ions into the concrete interior. Electrical pulses were utilized as an external electric field source by Huang et al. [21,22,23], and control experiments were conducted by immersing cement specimens with identical mix proportions in sulfate solutions with and without electric-field-accelerated attack. The results indicated that the penetration rate of SO_4_^2−^ into the cement mortar was significantly accelerated by electrical pulses, leading to the formation of more AFt and an increased depth of sulfate attack. Under the electric field generated by stray current, the directional migration of anions and cations within the pore solution of cement-based materials occurs, which accelerates the massive leaching of Ca^2+^ and OH^−^. This subsequently leads to the decomposition of Ca(OH)_2_ and C-S-H gels, as well as the severe degradation of the pore structure [24,25,26]. However, the reaction mechanisms and hydration products of geopolymers are distinctly different from those of traditional cement. Currently, very little research has been conducted on the leaching characteristics and deterioration laws of geopolymers under the action of stray current. In particular, under the coupled action of stray current and environmental aqueous media (soft water), the stability of the internal gel phase, the evolution of pore solution alkalinity, and the microstructural deterioration mechanism of geopolymers remain unclear. Furthermore, the macroscopic performance of FAG is affected by the coupled leaching of stray current and soft water, the fundamental cause of which lies in its impact on microscopic properties.

In Portland cement-based systems, the electric field promotes the directional migration of Ca^2+^ and OH^−^, thereby accelerating the dissolution of Ca(OH)_2_, the decalcification of C–S–H, and pore-structure deterioration. However, this calcium-dominated deterioration mechanism cannot be directly applied to low-calcium fly-ash geopolymers. The principal binding phase of the latter is N–A–S–H gel, in which alkali cations compensate for the negative charge generated by AlO_4_ tetrahedra. Therefore, electric-field-induced depletion of OH^−^ and mobile alkali species may disturb the chemical environment and structural stability of the aluminosilicate network through a mechanism fundamentally different from the decalcification of Portland cement hydrates.

Despite the above progress, several knowledge gaps remain. First, most durability studies on geopolymers have considered individual chemical or environmental actions, whereas the interaction between an external DC electric field and soft-water leaching remains insufficiently understood. Second, the deterioration mechanism established for calcium-rich cementitious systems does not adequately explain the response of an N–A–S–H-dominated geopolymer to OH^−^ and alkali depletion. Third, the causal relationship among pore-solution alkalinity loss, gel degradation, crystalline-phase stability, and pore-structure deterioration has not been systematically established under coupled stray-current and soft-water exposure. Finally, the roles of key mixture parameters in regulating this coupled deterioration pathway remain unclear.

In this study, the microscopic leaching mechanism of FAG is deeply investigated by simulating an aggressive environment involving the coupled action of DC stray current and soft water. The advancement of the present study over previous work lies in four aspects: (1) a coupled electrochemical–aqueous exposure condition is considered rather than a conventional single-agent environment; (2) the OH^−^ concentration in the external solution and the alkalinity of the internal pore solution are jointly evaluated; (3) solution-chemistry changes are correlated with XRD, FTIR, SEM, and MIP observations to establish a deterioration pathway from OH^−^ depletion to N–A–S–H degradation and pore coarsening; and (4) the effects of the water-to-binder ratio, sodium silicate modulus, and alkali dosage are interpreted mechanistically. This work, therefore, extends current knowledge of geopolymer durability from passive chemical attack to electrochemically accelerated leaching in underground environments.

## 2. Materials and Methods

### 2.1. Experimental Materials

**Figure 1:**
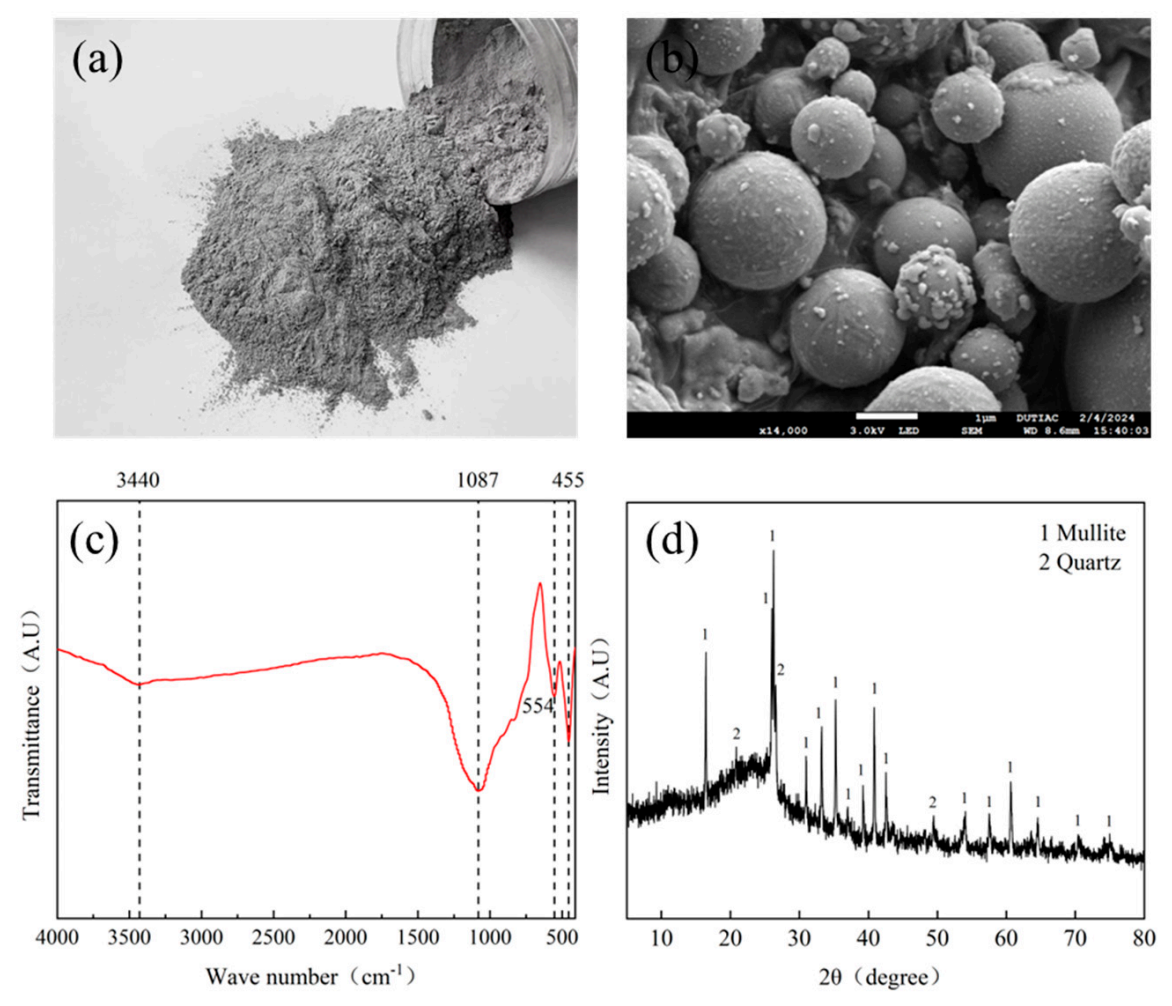
Fly ash. (**a**) Macro chart; (**b**) SEM microstructure; (**c**) FTIR analysis; (**d**) XRD phase analysis.

Class I fly ash, sourced from the Huaneng Power Plant in Qingdao, Shandong Province, was utilized in this study. Its physical properties and chemical composition are presented in Table 1. Infrared absorption spectroscopy and phase analysis of the fly ash were conducted using a Nicolet iS10 Fourier transform infrared (FTIR) spectrometer (Thermo Fisher Scientific, Waltham, MA, USA) and a D8 Advance X-ray diffractometer (XRD) (Bruker, Ettlingen, Germany), respectively. The corresponding test results are illustrated in Figure 1.

The fly ash contained 53.86 wt.% SiO_2_, 29.45 wt.% Al_2_O_3_, and only 3.71 wt.% CaO, indicating a low-calcium aluminosilicate precursor. Based on the bulk oxide composition, the approximate atomic Si/Al ratio was 1.55. SEM observations showed that the precursor consisted predominantly of spherical particles with attached finer particles, a morphology that is generally favorable for flowability but may produce heterogeneous reaction degrees among particles.

The broad FTIR band near 1087 cm^−1^ is associated with Si–O–Si and Si–O–Al vibrations in the aluminosilicate structure. The XRD pattern contains identifiable quartz and mullite peaks superimposed on a broad diffuse background, indicating the coexistence of crystalline residual phases and an amorphous or poorly crystalline aluminosilicate fraction. The latter provides the principal reactive component for alkali activation, whereas quartz and mullite are expected to remain comparatively less reactive.

Sodium silicate (Na_2_O·nSiO_2_) was supplied by Linyi Lusen Chemical Co., Ltd. (Linyi, China), and its primary technical specifications are presented in Table 2. Sodium hydroxide (NaOH) was manufactured by Tianjin Kemiou Chemical Reagent Co., Ltd. (Tianjin, China). Deionized water, sourced from Dalian Jinke Chemical Reagent Co., Ltd. (Dalian, China), was utilized throughout all experiments.

The sodium silicate modulus (Ms) is defined as the molar ratio of SiO_2_ to Na_2_O within the sodium silicate. The calculation method for this modulus, as stipulated by the Chinese National Standard Industrial sodium silicate (GB/T 4209-2022) [27], is expressed in Equation (1).

(1) $$\mathrm{Ms}=\frac{\omega_{SiO_{2}}}{\omega_{Na_{2}O}}\times 1.032,$$

where $\omega_{SiO_{2}}$ and $\omega_{Na_{2}O}$ represent the mass fractions of SiO_2_ and Na_2_O in the sodium silicate, respectively.

To enhance the solubility and chemical reactivity of the sodium silicate, and to reduce its viscosity, the modulus of the sodium silicate utilized in this experiment was restricted to no more than 2.0 [28]. The viscosity of the solution was significantly decreased by the incorporation of sodium hydroxide, thereby facilitating a complete reaction. The corresponding chemical equation is expressed in Equation (2).

(2) $$Na_{2}SiO_{3}+2\mathrm{NaOH}\to Na_{2}O\cdot \mathrm{Si}O_{2}+Na_{2}O+H_{2}O$$

Based on an analysis of Equations (1) and (2), the mass fraction of NaOH to be incorporated into the original sodium silicate to achieve the designed modulus can be calculated using Equations (3) and (4). According to the aforementioned calculation method, the mass fractions of NaOH incorporated during the preparation of sodium silicates with various moduli utilized in this study are presented in Table 3.

(3) $$\omega_{Na_{2}O}^{\prime }=\frac{\mathrm{Ms}\cdot \omega_{Na_{2}O}}{Ms^{\prime }}$$

(4) $$\omega_{\mathrm{NaOH}}=\frac{40\left(\omega_{Na_{2}O}^{\prime }-\omega_{Na_{2}O}\right)}{31}$$

where Ms and $Ms^{\prime }$ are the moduli of the original and target sodium silicates, respectively; $\omega_{Na_{2}O}$ and $\omega_{N_{a_{2}O}}^{\prime }$ are the mass fractions of Na_2_O in the original and target sodium silicates, respectively; and $\omega_{\mathrm{NaOH}}$ is the mass fraction of the incorporated NaOH.

### 2.2. Mix Proportions

To systematically investigate the microscopic mechanisms underlying the resistance of FAG to the coupled leaching of stray current and soft water, the group exhibiting the lowest compressive strength loss rate was selected as the reference group based on preliminary orthogonal test results [29]. The mix proportion for this reference group was established as follows: a water-to-binder ratio (W/C) of 0.30, a sodium silicate modulus (Ms) of 1.2, and an alkali dosage (A/F, defined as the mass ratio of Na_2_O to fly ash) of 6%.

The selection of the three mixture parameters was based on their distinct roles in geopolymer formation and subsequent ion transport. W/C controls the amount of free water and strongly influences the formation and connectivity of capillary pores; consequently, it affects the pathways available for ionic migration and leaching. Ms represents the SiO_2_/Na_2_O ratio of the sodium silicate activator and regulates the balance between soluble silicate availability, solution alkalinity, activator viscosity, and gel polymerization. A/F determines the amount of Na_2_O relative to fly ash and, therefore, affects precursor dissolution, charge compensation within the N–A–S–H gel, and the initial alkaline reserve of the pore solution.

The ranges W/C = 0.28–0.34, Ms = 1.0–1.6, and A/F = 4–7% were selected around the mechanically favorable region identified in the preliminary orthogonal study [29]. The selected lower and upper levels represent, respectively, relatively dense/low-water to more porous/high-water systems, highly alkaline/low-modulus to more silicate-rich activators, and relatively low to relatively high alkali availability. A single-factor design was adopted so that the effect of each parameter on OH^−^ loss, gel stability, and pore-structure evolution could be isolated while the remaining parameters were held constant. The specific experimental mix proportions designed for this mechanistic investigation are detailed in Table 4.

### 2.3. Experimental Methods

**Figure 2:**
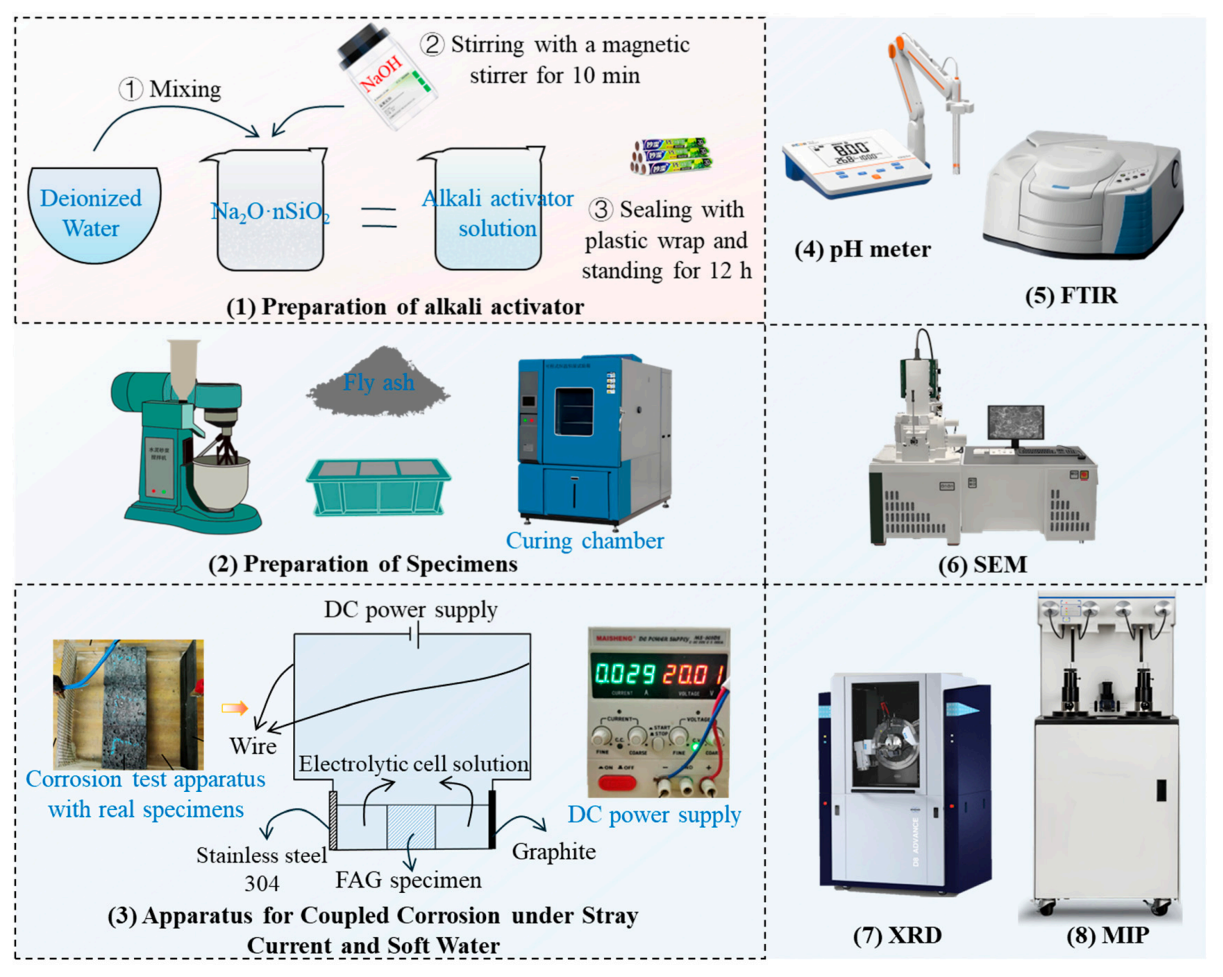
Experimental processes.

(1) Preparation of alkali activator: The required quantities of sodium silicate, water, and sodium hydroxide were calculated according to the designed mix proportions. Initially, the sodium silicate and water were mixed in a beaker and stirred uniformly using a glass rod. Subsequently, the sodium hydroxide was added, and the mixture was stirred with a magnetic stirrer for 10 min until the prepared activator became clear and transparent. Upon the completion of stirring, the activator was placed indoors and allowed to stand for at least 12 h. During both the magnetic stirring and the standing processes, the beaker was sealed with plastic wrap to prevent the evaporation of water.

(2) Preparation of specimens: The preparation of the fly ash geopolymer (FAG) was conducted only after the activator had finished standing. A JJ-5 cement mortar mixer, manufactured by Wuxi Jianyi Instrument & Machinery Co., Ltd. (Wuxi, China), was utilized for blending the paste. First, the mixing blade and bowl were moistened with a damp cloth, ensuring no excess moisture remained, after which the activator and the pre-weighed fly ash were introduced. The mixed paste was cast into 40 mm × 40 mm × 40 mm molds in two stages. The molds were initially filled to half their capacity and vibrated on a vibrating table for 30 s. Subsequently, the remaining half was filled, followed by another 30 s of vibration. Excess paste on the mold surface was then scraped off, and the molds were covered with plastic wrap. To significantly enhance the early strength development rate of the FAG while preventing microstructural expansion and cracking caused by excessive temperatures, a previously optimized high-temperature curing regime at 80 °C was adopted in this study. The specific procedure was as follows: the surfaces of the fresh specimens were covered with plastic wrap, sealed entirely within ziplock bags, and placed into a high-temperature curing oven at 80 °C. Demolding was performed after 6 h of high-temperature curing. The demolded specimens were re-sealed in ziplock bags and returned to the oven until a total of 24 h of high-temperature curing was completed. Following this, the specimens were transferred to a standard curing environment and cured for up to 28 d to ensure stable strength for the subsequent coupled leaching tests and micro-mechanistic characterization.

(3) Apparatus for coupled leaching under stray current and soft water: The apparatus utilized for the coupled leaching under stray current and soft water in this study is illustrated in Figure 2. An acrylic mold with internal dimensions of 120 mm × 120 mm × 40 mm was fabricated. Graphite and 304 stainless steel were employed as the anode and cathode materials, respectively, and were connected to the positive and negative terminals of a direct current (DC) power supply via copper wires. Deionized water was utilized as the electrolytic cell solution. The entire leaching apparatus can be regarded as an electrolytic cell, wherein an oxidation reaction, as expressed in Equation (5), occurs at the anode (the electrode connected to the positive terminal of the DC power supply) during the leaching process. It is noteworthy that an inert electrode should be utilized for the anode; the use of metal electrodes might result in the discharge of the electrode material, thereby interfering with the experiment. Therefore, graphite was adopted as the anode electrode in this study. At the cathode (the electrode connected to the negative terminal of the DC power supply), a reduction reaction, as expressed in Equation (6), occurs during the leaching process. Since a reduction reaction takes place on the cathode side, the discharge of the electrode material does not need to be considered; hence, 304 stainless steel was utilized as the cathode electrode.

For the microstructural investigation, a constant DC voltage of 20 V was applied for exposure periods of up to 90 d. The distance between the electrodes was 110 mm, the immersion depth of the specimens was 300 mm. These parameters should be reported to improve the reproducibility of the accelerated test. The laboratory system was designed as a controlled accelerated exposure rather than as a direct reproduction of a particular subway structure. The imposed DC electric field represents the directional migration of ionic species induced by stray current, whereas deionized water provides a simplified low-mineralization soft-water environment. Replacing the external solution every 7 d maintained the concentration gradient between the FAG pore solution and the external aqueous medium and avoided suppression of further leaching by accumulated OH^−^. This periodic renewal also represents, in a simplified manner, the continuous replenishment of groundwater around underground structures. It should be emphasized that the constant 20 V condition is more severe and less fluctuating than the stray-current environment generally experienced by an in-service structure. Furthermore, natural groundwater may contain Ca^2+^, Mg^2+^, Cl^−^, SO_4_^2−^, and dissolved carbon species that are absent from the deionized-water system. Therefore, the present test is intended to accelerate and isolate the coupled electromigration–leaching mechanism and to compare mixture parameters under identical conditions; it is not intended to establish a direct laboratory-to-field time conversion or predict service life.

The applied voltage of 20 V was selected from the exposure levels evaluated in the authors’ previous study [29], which included soft-water-only, 20 V, and 40 V conditions. In the present investigation, 20 V was used as an intermediate accelerated condition to produce measurable but progressive deterioration over the 90 d microscopic observation period. This voltage should not be interpreted as the actual stray voltage acting on a specific subway structure. Rather, it represents a controlled laboratory acceleration boundary condition. Field stray-current exposure varies with time and location and is influenced by rail-to-earth leakage, structural and soil resistivity, groundwater chemistry, electrical continuity, and current-return paths. Consequently, the laboratory exposure duration cannot be directly converted into an equivalent field service period.

(5) $$4OH^{-}-4e^{-}\to 2H_{2}O+O_{2}↑$$

(6) $$2H_{2}O+2e^{-}\to 2OH^{-}+H_{2}↑$$

(4) Measurement of pore solution alkalinity and OH^−^ leaching amount: Following the completion of the compressive strength tests, uniform sampling was conducted on the crushed specimens. The samples were immersed in an adequate amount of absolute ethanol for 48 h to terminate hydration. The samples were then retrieved, and the residual absolute ethanol on their surfaces was removed using absorbent paper. Subsequently, they were placed in a vacuum drying oven at 60 °C for 24 h to ensure the complete evaporation of the ethanol. The dried samples were thoroughly ground using a mortar. Once reduced to powder, the samples were sieved through a 75 μm sieve, and the powder passing through the sieve was collected. Because the powder might absorb water vapor from the air during the grinding and sieving processes, the samples were placed back into the vacuum drying oven at 60 °C for another 24 h to guarantee the complete evaporation of any absorbed moisture, thereby completing the sample preparation. The prepared samples were mixed with deionized water at a mass ratio of 1:10, agitated for 48 h, and then allowed to stand for 24 h. The supernatant was extracted, and its pH value was measured using a PHS-3C pH meter (Shanghai INESA Scientific Instrument Co., Ltd., China). Prior to testing, a three-point calibration was performed using standard buffer solutions with pH values of 4.00, 6.86, and 9.18. For each group of solutions to be tested, the electrode was rinsed with deionized water and dried with absorbent paper before measurement.

During the leaching process of the FAG, the leaching of OH^−^ is accelerated by the stray current, resulting in a significant increase in the OH^−^ concentration within the electrolytic cell solution. Therefore, the electrolytic cell solution was periodically replaced to prevent the inhibition of further OH^−^ leaching caused by excessively high OH^−^ concentrations. According to the experimental design, the electrolytic cell solution was replaced with deionized water every 7 d. Because the current intensities of the electrolytic cells varied among the different groups, leading to slight differences in the solution volumes, the collected replacement solutions were made up to a constant volume of 500 mL prior to pH testing. The pH value of the pore solution was converted into the OH^−^ concentration using Equation (7), which was subsequently utilized to characterize the pore solution alkalinity of the FAG.

(7) $$c_{\left(OH^{-}\right)}=10^{\left(pH-14\right)}$$

where $c_{OH^{-}}$ and pH represent the molar concentration of OH^−^ and the pH value of the pore solution, respectively.

The present solution-chemistry analysis focused on pH-derived OH^−^ concentration as an indicator of pore-solution alkalinity loss. Dissolved Na, Si, and Al were not quantified. Consequently, the present measurements cannot independently resolve alkali-cation migration, aluminosilicate dissolution, or the elemental mass balance of the leaching process.

(5) FTIR testing: A Nicolet iS10 Fourier transform infrared (FTIR) spectrometer (Thermo Fisher Scientific, USA) was utilized for the FTIR analysis. The tested wavenumber range was 400–4000 cm^−1^, with a resolution of 4 cm^−1^. The variations in the characteristic peaks of functional groups, such as Al–O and Si–O, in the fly ash geopolymer before and after the coupled leaching of stray current and soft water were determined.

(6) SEM testing: A JSM-7900F Plus field emission scanning electron microscope (SEM) (JEOL Ltd., Tokyo, Japan) was employed for the microstructural analysis. The magnification utilized during the observation ranged from 3000× to 20,000×. The micro-morphology of the fly ash geopolymer before and after the coupled leaching of stray current and soft water was observed.

(7) XRD testing: A D8 Advance X-ray diffractometer (XRD) (Bruker, Germany) was utilized for the phase analysis. During the test, the dwell time per step was 0.1 s, the step size was 0.02°, and the scanning range was 5–80°. Qualitative testing was conducted on the fly ash geopolymer before and after the coupled leaching of stray current and soft water to analyze the phase transformations induced by the leaching process.

(8) MIP testing: An AutoPore IV9500 mercury intrusion porosimeter (Micromeritics Instrument Corp., Norcross, GA, USA) was utilized for the pore structure analysis. The tested pore diameter range was 5 nm–1000 μm, and the maximum applied pressure was 228 MPa. The pore structure of the FAG before and after the coupled leaching of stray current and soft water was characterized. Based on the pore type, the pores within cementitious materials can be classified into gel pores (diameter < 10 nm), transition pores (10 nm ≤ diameter < 100 nm), capillary pores (100 nm ≤ diameter < 1000 nm), and macropores (diameter ≥ 1000 nm). Furthermore, according to their degree of harmfulness, they can be categorized into harmless pores (diameter < 50 nm), less harmful pores (50 nm ≤ diameter < 200 nm), and highly harmful pores (diameter ≥ 200 nm) [30,31,32].

The pH, calculated OH^−^ concentration, cumulative OH^−^ release, total porosity, most probable pore diameter, and pore-size fractions were treated as quantitative indicators. By contrast, XRD was used for phase identification rather than quantitative phase analysis because no internal standard or Rietveld refinement was employed. FTIR was used to evaluate relative changes in characteristic bands, and SEM was used to identify representative morphological features. Therefore, XRD, FTIR, and SEM observations are interpreted qualitatively or semi-quantitatively and are not used to calculate absolute gel contents or phase-volume fractions.

## 3. Results and Discussion

### 3.1. Time Effect of Coupled Stray Current and Soft Water Leaching

**Figure 3:**
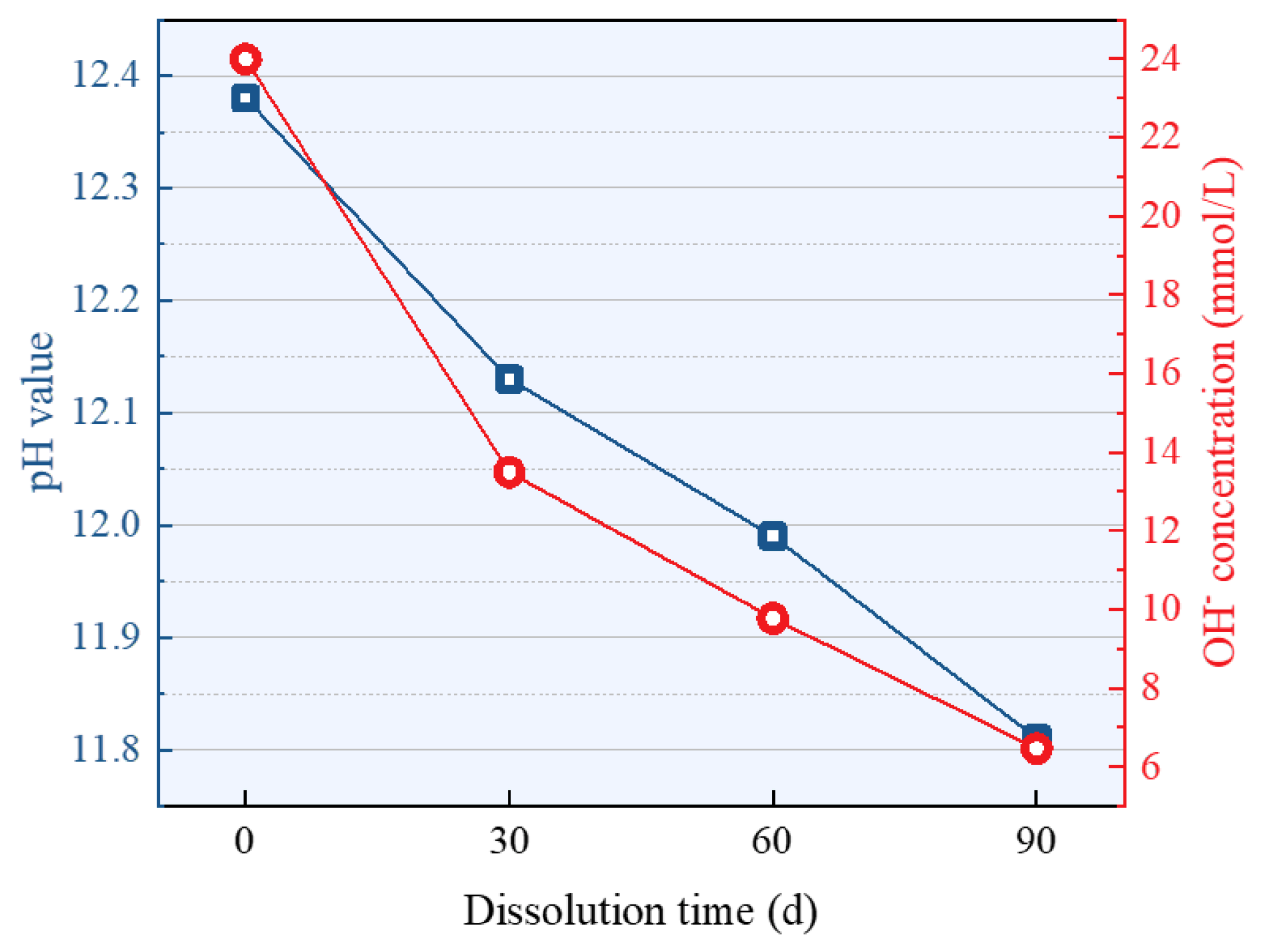
Changes in pH value and OH^−^ concentration.

The microstructural degradation of the FAG induced by the coupled action of stray current and soft water is a dynamic, time-dependent process. This is primarily manifested by the loss of alkalinity, phase degradation, and the deterioration of the pore structure. As illustrated in Figure 3, as the leaching time increased, the pH value of the pore solution in the reference group FAG (W/C = 0.30, Ms = 1.2, A/F = 6%) gradually decreased from 12.38 to 11.81 at 90 d. Concurrently, the OH^−^ concentration of the pore solution was significantly reduced from 23.99 mmol/L to 6.46 mmol/L, and a decelerating trend in the reduction rate was observed with prolonged leaching time.

**Figure 4:**
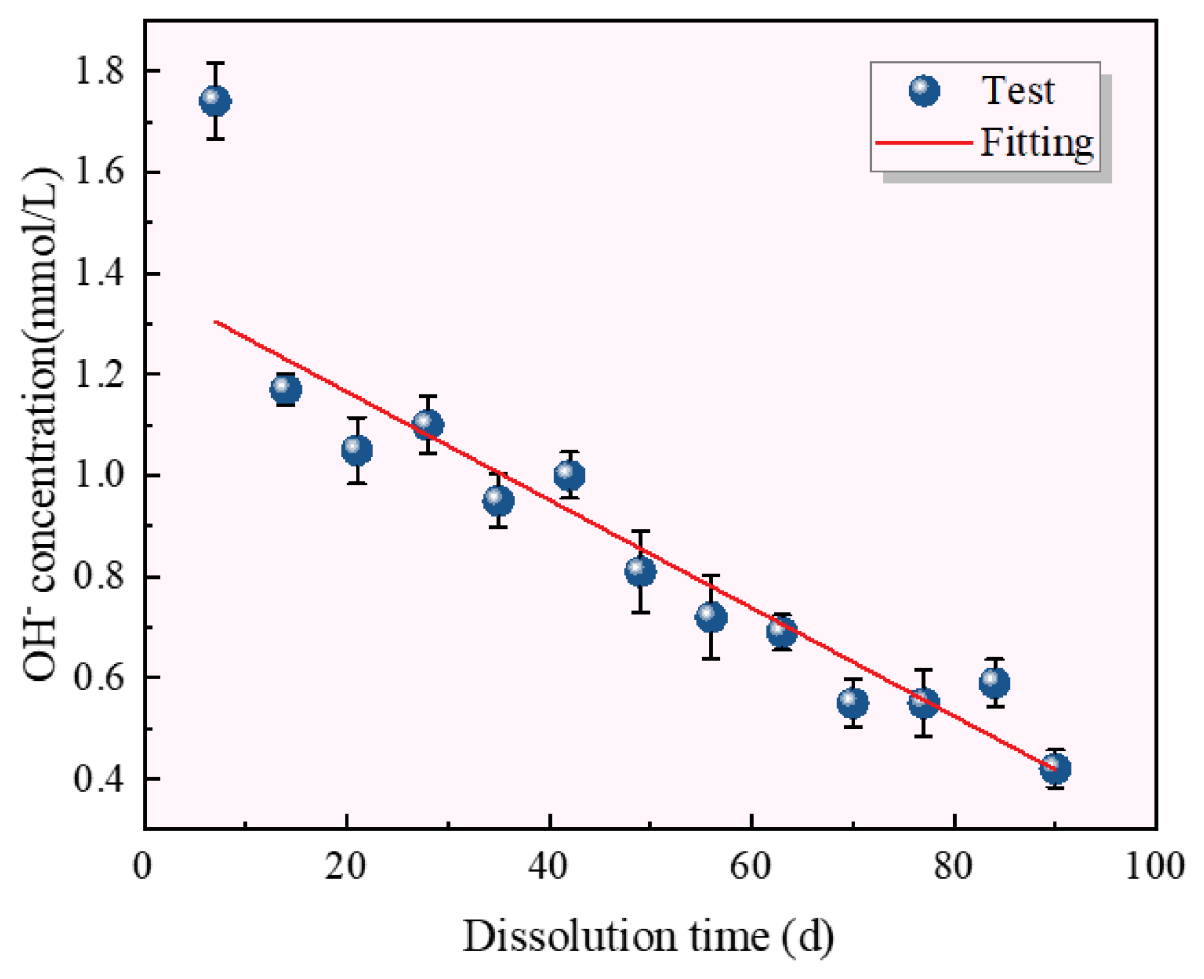
Comparison between linear fitting and test results.

The electrolytic cell solution was replaced every 7 d during the leaching process. As depicted in Figure 4, despite slight fluctuations, an overall decreasing trend in the OH^−^ concentration of the electrolytic cell solution was observed as the leaching time progressed. This indicates that the coupled leaching effect of the stray current and soft water is relatively pronounced during the early stages of leaching, whereas this coupled effect is gradually weakened over time. Based on the observation of the curve trend in Figure 4, a potential linear correlation between the OH^−^ concentration of the electrolytic cell solution and the leaching time was identified. Therefore, the linear correlation coefficient, r, between the two variables was calculated using Equation (8).

(8) $$r=\frac{\sum_{1}^{n}\left(t_{i}-\bar{t})(c_{i}-\bar{c}\right)}{\sqrt{\sum_{1}^{n}{\left(t_{i}-\bar{t}\right)}^{2}}\sqrt{\sum_{1}^{n}{\left(c_{i}-\bar{c}\right)}^{2}}}$$

where $t_{i}$ and $\bar{t}$ represent the i-th leaching time and the average leaching time, respectively; and $c_{i}$ and $\bar{c}$ represent the OH^−^ concentration of the electrolytic cell solution corresponding to the i-th leaching time and the average OH^−^ concentration of the electrolytic cell solution, respectively.

The calculated correlation coefficient yielded r=−0.93. Generally, a linear correlation is considered to exist between two variables when |r|≥0.5, and a strong linear correlation is considered to exist when |r|≥0.7. Therefore, it can be concluded that a strong linear correlation exists between the leaching time and the OH^−^ concentration of the electrolytic cell solution. The linear relationship equation between the two variables was derived utilizing the least squares method, with the calculation procedure detailed in Equations (9)–(11).

(9) $$\hat{b}=\frac{\sum_{1}^{n}t_{i}c_{i}-n\bar{t}\bar{c}}{\sum_{1}^{n}t_{i}^{2}-n{\bar{t}}^{2}}$$

(10) $$\hat{a}=\bar{c}-\hat{b}\bar{t}$$

(11) $$c=\hat{b}t+\hat{a}$$

where $t_{i}$ is the i-th leaching time; $\bar{t}$ is the average leaching time; $c_{i}$ is the OH^−^ concentration of the electrolytic cell solution corresponding to the i-th leaching time; and $\bar{c}$ is the average OH^−^ concentration of the electrolytic cell solution.

The calculation yielded $\hat{b}=-0.0120$, $\hat{a}=1.4623$; consequently, the linear relationship between the leaching time and the OH^−^ concentration of the electrolytic cell solution for the reference group is expressed as Equation (12).

(12) c=−0.0120t+1.4623

It should be noted that the effects of the testing cycle and the solution volume are not considered in Equation (12). To determine the relationship between the molar amount of leached OH^−^ and the leaching time, a testing cycle of 7 d and an electrolytic cell solution volume of 0.5 L were taken into account; consequently, the equation governing the relationship between the molar amount of leached OH^−^ and the leaching time was derived using Equation (13). Based on the calculation from Equation (13), the relationship between the molar amount of leached OH^−^ and the OH^−^ concentration of the electrolytic cell solution is expressed as Equation (14).

(13) $$n=\frac{0.5c}{7}$$

(14) n=−0.00086t+0.10445

where n is the molar amount of leached OH^−^; c represents the OH^−^ concentration of the electrolytic cell solution calculated via Equation (12); t denotes the leaching time; and n is the molar amount of leached OH^−^ corresponding to the leaching time.

During the coupled leaching process involving stray current and soft water, a linear relationship was identified between the OH^−^ concentration of the electrolytic cell solution and the leaching time. The linear equation governing these two variables was derived utilizing the least squares method, and it was subsequently converted into a relationship expressing the molar amount of leached OH^−^ per unit time as a function of the leaching time. The results indicate that the coupled leaching rate induced by stray current and soft water decelerates as the leaching time progresses, and a linear relationship is maintained between the leaching rate and the leaching time. It should be emphasized that this linear relationship is an empirical description of the OH^−^ concentration measured in the periodically renewed solution over the investigated time range, rather than a universal diffusion-controlled kinetic law. The periodic renewal of the external solution and the contribution of electric-field-driven ion migration prevent direct extraction of an intrinsic diffusion coefficient from the present dataset.

**Figure 5:**
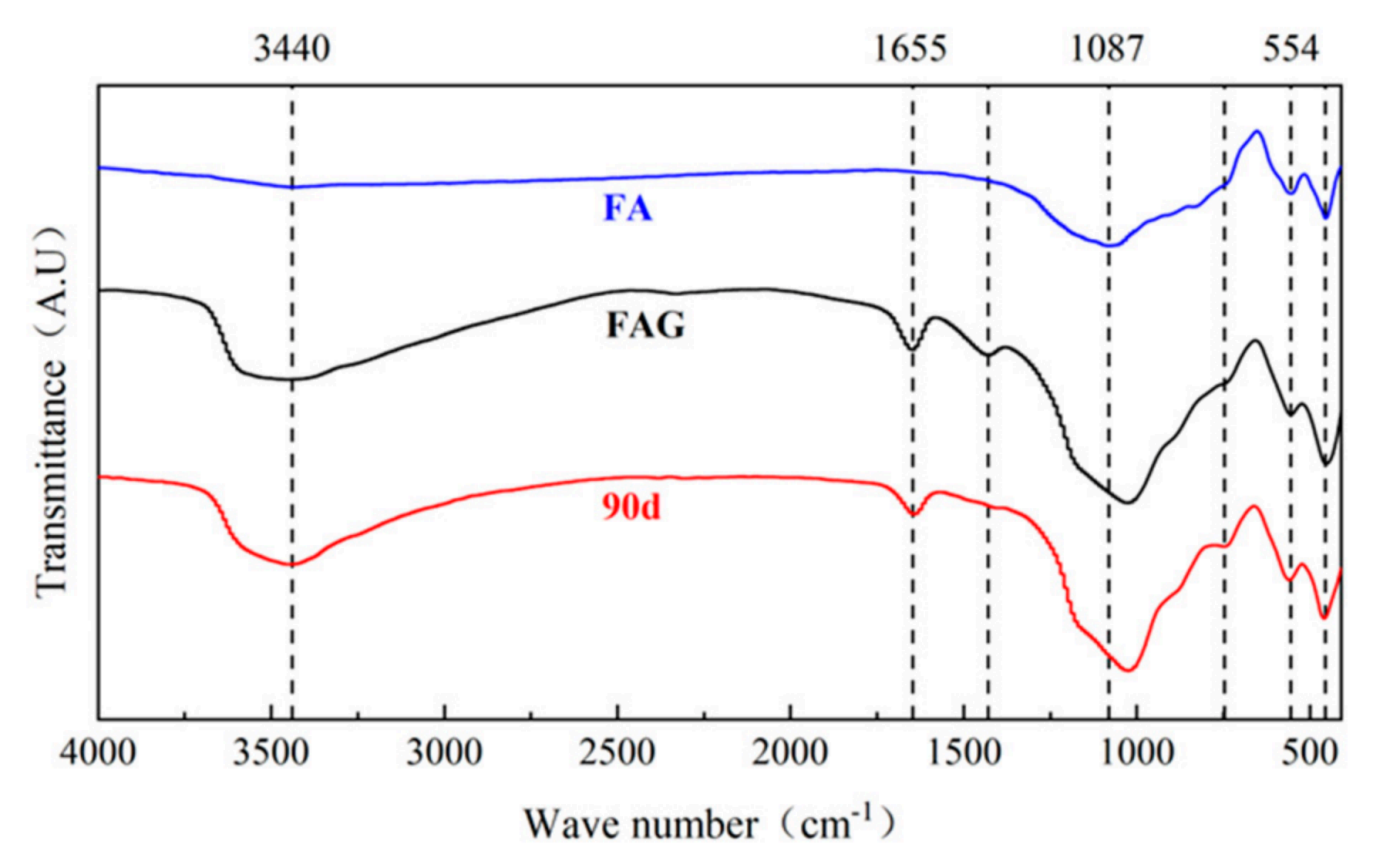
Infrared spectrum of fly ash and FAG before and after corrosion.

The FTIR spectra of the raw fly ash and the FAG before and after leaching are presented in Figure 5. The FTIR analysis demonstrates that after 90 d of leaching, the intensities of both the absorption peak at 1027 cm^−1^, corresponding to the stretching vibrations of Si–O–Si and Si–O–Al, and the bending vibration peak at 459 cm^−1^ within the FAG are diminished. This change is consistent with an alteration or partial degradation of the N–A–S–H-related bonding environment during the leaching process. Furthermore, the near-complete disappearance of the band situated around 1433 cm^−1^ is consistent with the observed reduction in the alkaline pore-solution environment. Because Na^+^ release was not directly measured, no quantitative conclusion regarding Na migration is drawn from the present FTIR results.

**Figure 6:**
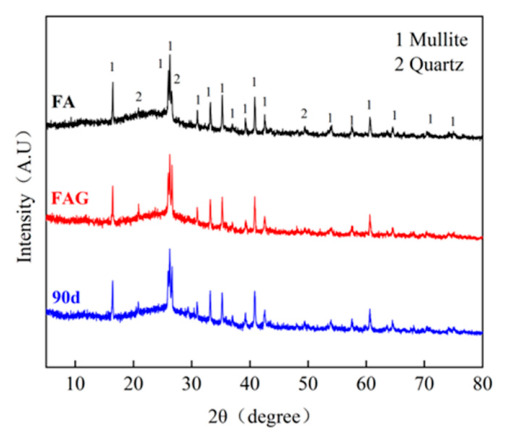
X-ray diffraction pattern of fly ash and FAG before and after corrosion.

The XRD patterns of the raw fly ash and the FAG before and after leaching are illustrated in Figure 6. As depicted in the figure, minimal fluctuation is observed in the broad diffuse peak located between 15° and 30° during the coupled leaching process involving stray current and soft water. This suggests that only minor phase transformations occur within the gel over the course of the leaching process. The intensities of the primary diffraction peaks corresponding to the quartz and mullite crystalline phases remain virtually unchanged, and no new diffraction peaks are detected. The persistence of the major quartz and mullite peaks suggests that no pronounced transformation of these crystalline phases was detectable by the qualitative XRD analysis. However, because quantitative phase refinement was not performed, small changes in their phase fractions cannot be excluded.

**Figure 7:**
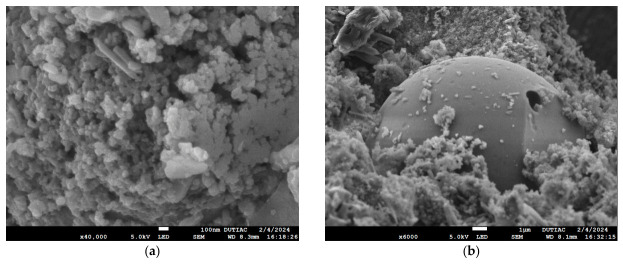
Microstructure of fly ash geopolymer. (**a**) N-A-S-H gel; (**b**) Unreacted fly ash particles.

The SEM images of the FAG prior to leaching are presented in Figure 7. The flocculent gel observed in Figure 7a is identified as N-A-S-H gel. The formation process of this gel proceeds as follows: the aluminosilicate glass phase within the fly ash is depolymerized under the action of OH^−^, forming oligomeric [SiO_4_] and [AlO_4_] species. These species subsequently undergo a polymerization reaction with the oligomeric [SiO_4_] introduced by the activator to generate a gel. Na^+^ then enters the interior of the colloid to balance the negative charge generated by the aluminate tetrahedra, ultimately forming the N-A-S-H gel [33]. Furthermore, as demonstrated in Figure 7b, incompletely reacted fly ash particles are present within the FAG. These particles are tightly encapsulated by the N-A-S-H gel, and their surfaces remain highly smooth, indicating a minimal degree of reaction with the activator. This phenomenon is attributed to the fact that the N-A-S-H gel, generated from the reaction of certain fly ash particles, adheres to the surfaces of other fly ash particles, thereby hindering further reaction between OH^−^ and the remaining fly ash particles [34].

**Figure 8:**
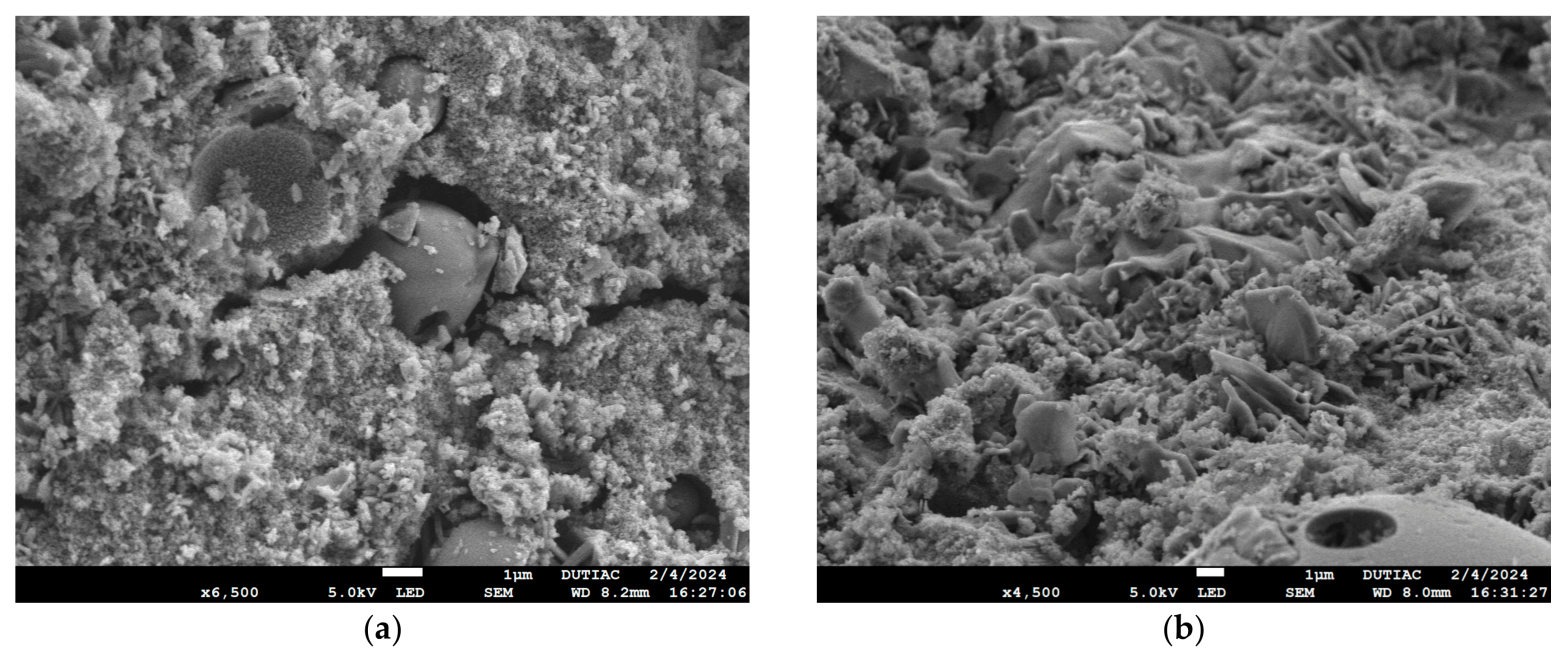

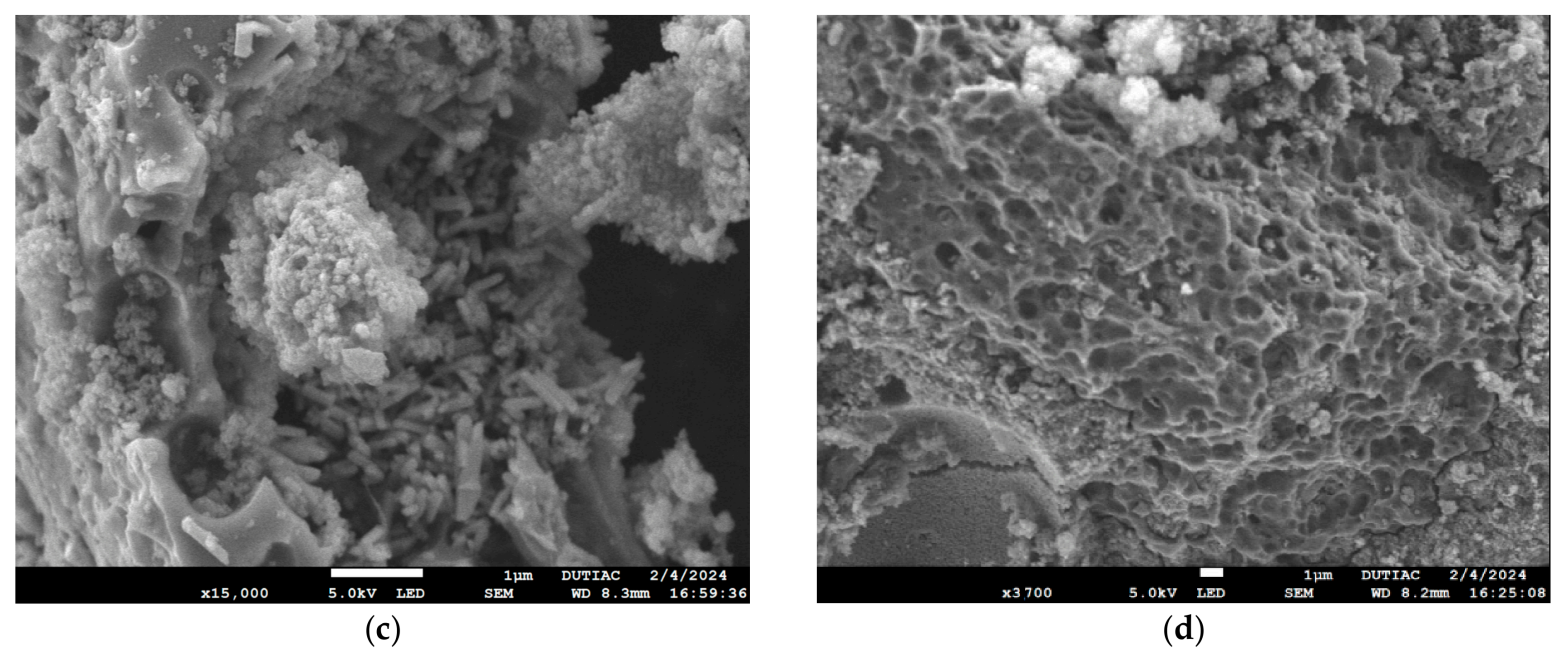
Microstructure of fly ash geopolymer at different corrosion times (**a**–**d**).

The SEM images of the FAG subjected to a 20 V voltage for various leaching times are presented in Figure 8. As demonstrated in Figure 8a, incompletely reacted fly ash particles remain present within the FAG following the coupled leaching of stray current and soft water. However, in contrast to the FAG prior to leaching, these fly ash particles are observed to be detached from the gel matrix, or massive pores are generated at the interface between the particles and the gel. This indicates that the binding effect of the gel matrix on the overall system is weakened by the leaching process. Furthermore, as illustrated in Figure 8b,c, the N-A-S-H gel is degraded after leaching. Concurrently, owing to the deterioration of the gel phase, the originally encapsulated crystalline phases are exposed, which predominantly include acicular mullite, short-rod-like quartz, and plate-like albite. As depicted in Figure 8d, with the progression of the leaching process, the N-A-S-H gel is further destroyed, and a porous structure is developed within the interior of the FAG.

**Figure 9:**
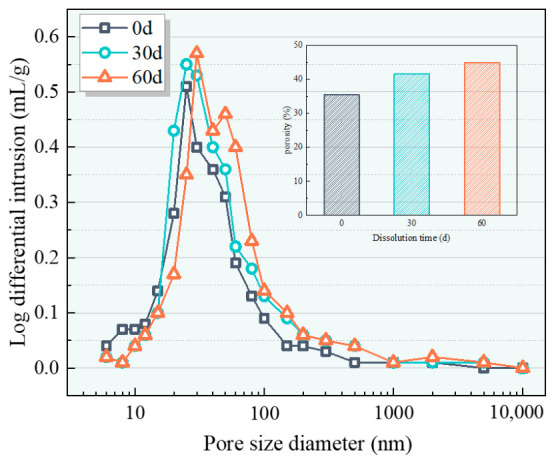
Pore size distribution and porosity of benchmark group.

As illustrated in Figure 9, the MIP analysis demonstrates that after 30 d of leaching, the quantity of gel pores smaller than 10 nm is substantially reduced, whereas the quantity of harmful pores ranging from 50 to 300 nm is drastically increased. With the progression of time, the most probable pore diameter is enlarged from an initial 26.5 nm to 33.2 nm at 90 d. Concurrently, the total porosity of the specimens is significantly increased from 35.5% prior to leaching to 44.9% at 90 d. This indicates that the deterioration of the macroscopic strength of the FAG is collectively induced by the degradation of the gel phase and the proliferation of harmful pores [35,36,37].

The coupled deterioration process can be interpreted as an electromigration–dealkalization–gel degradation–pore coarsening sequence. The connected pore solution provides the principal transport pathway for ionic species. Under the imposed DC electric field, charged species undergo directional migration in addition to ordinary diffusion. The present measurements directly demonstrate the depletion of OH^−^ from the FAG pore solution. Although Na^+^ migration was not directly measured, the loss of mobile alkali cations is chemically plausible because Na^+^ acts as a charge-balancing species for negatively charged AlO_4_ tetrahedra in the N–A–S–H network. Therefore, Na^+^ migration is considered a mechanistic inference rather than a directly measured result.

The reduction in OH^−^ concentration lowers pore-solution alkalinity and changes the chemical equilibrium between the pore solution and the aluminosilicate gel. This condition may promote hydrolysis and depolymerization of Si–O–Al and Si–O–Si linkages and reduce the stability or continuity of the N–A–S–H binding network. The weakened gel is consistent with the reduced FTIR band intensity near 1027 and 459 cm^−1^ and with the detachment of residual fly-ash particles from the surrounding matrix observed by SEM.

As the binding gel deteriorates, interfacial voids and connected capillary pores develop. This explains the increases in porosity, most probable pore diameter, and harmful-pore fraction measured by MIP. By contrast, quartz and mullite are relatively stable residual crystalline phases and do not serve as the principal binding phase; therefore, their diffraction peaks remain largely unchanged even when the surrounding amorphous gel is degraded.

The decline in the interval OH^−^ concentration with increasing exposure time may be associated with the progressive depletion of readily mobile alkalis, the reduction in the concentration difference between the interior and exterior solutions, and the formation of a degraded surface layer that increases the effective transport path. These processes jointly weaken the apparent coupled leaching effect at later exposure stages.

### 3.2. Regulatory Effects of Mix Proportion Parameters on the Leaching Mechanism

#### 3.2.1. Regulatory Effect of the Water-to-Binder (W/C) Ratio

**Figure 10:**
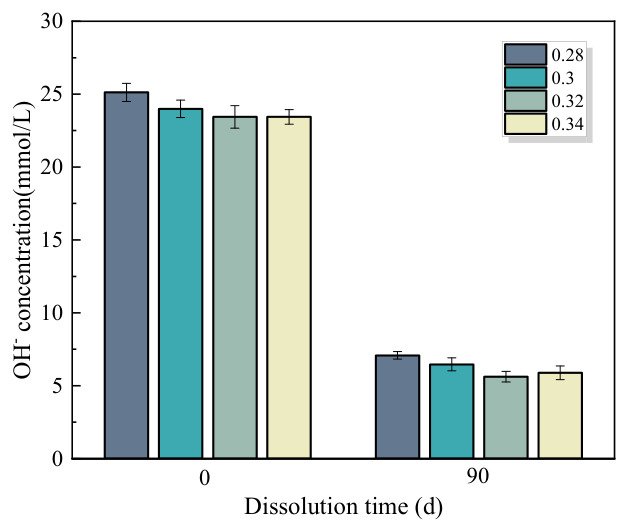
OH^−^ concentration pore solution of FAG with different W/C before and after corrosion.

**Figure 11:**
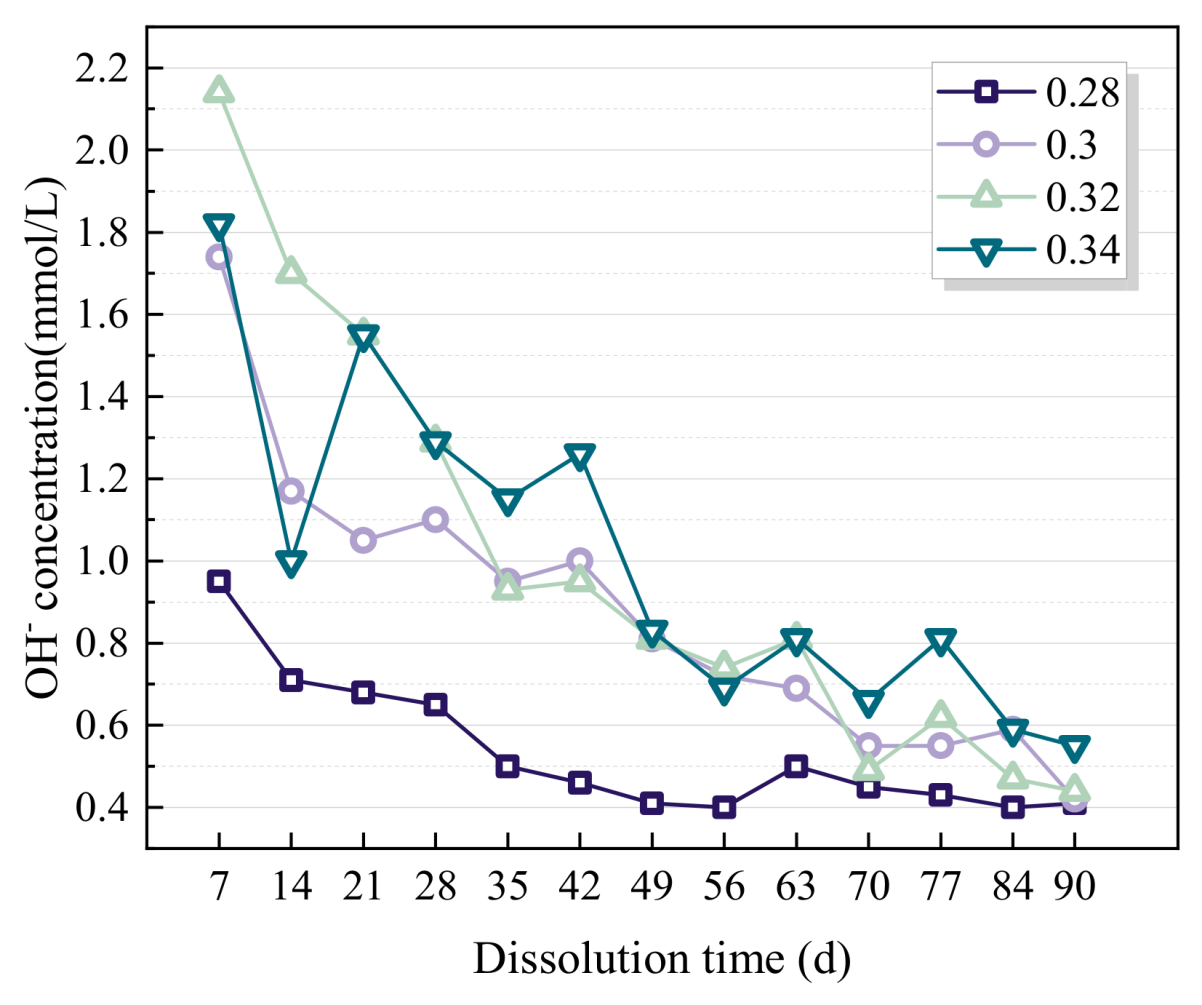
OH^−^ concentration in electrolytic cell solution of FAG with different W/C.

The OH^−^ concentrations in the pore solutions of the FAG with varying W/C ratios before and after leaching under an applied voltage of 20 V are presented in Figure 10. Both before and after leaching, the highest pore solution alkalinity is consistently observed in the FAG with a W/C ratio of 0.28. This indicates that a lower W/C ratio is conducive to maintaining the pore solution alkalinity of the FAG during the leaching process [38]. The variation in the OH^−^ concentration of the electrolytic cell solution with respect to leaching time for the groups with varying W/C ratios under an applied voltage of 20 V is illustrated in Figure 11. Overall, as the leaching time progresses, a reduction in the amount of OH^−^ leached per unit time is observed across all groups. This demonstrates that the coupled leaching effect induced by the stray current and soft water is attenuated as the leaching duration increases. At a lower W/C ratio, the coupled leaching effect of the stray current and soft water is relatively weak even during the initial stage of leaching. Conversely, at a higher W/C ratio, this coupled leaching effect is pronounced during the initial stage; however, a more significant attenuation of the coupling effect is exhibited as the leaching time progresses.

The minimum cumulative molar amount of leached OH^−^ is consistently recorded for the FAG group with a W/C ratio of 0.28 across all three designated leaching durations. At the leaching durations of 30 d and 60 d, the cumulative molar amount of leached OH^−^ initially increases and subsequently decreases with an ascending water-to-binder ratio. At 90 d, however, the cumulative molar amount of leached OH^−^ strictly increases with an increasing water-to-binder ratio. This indicates that the leaching of OH^−^ can be effectively inhibited by a lower W/C ratio, and this inhibitory effect becomes more pronounced during the later stages of the leaching process.

**Figure 12:**
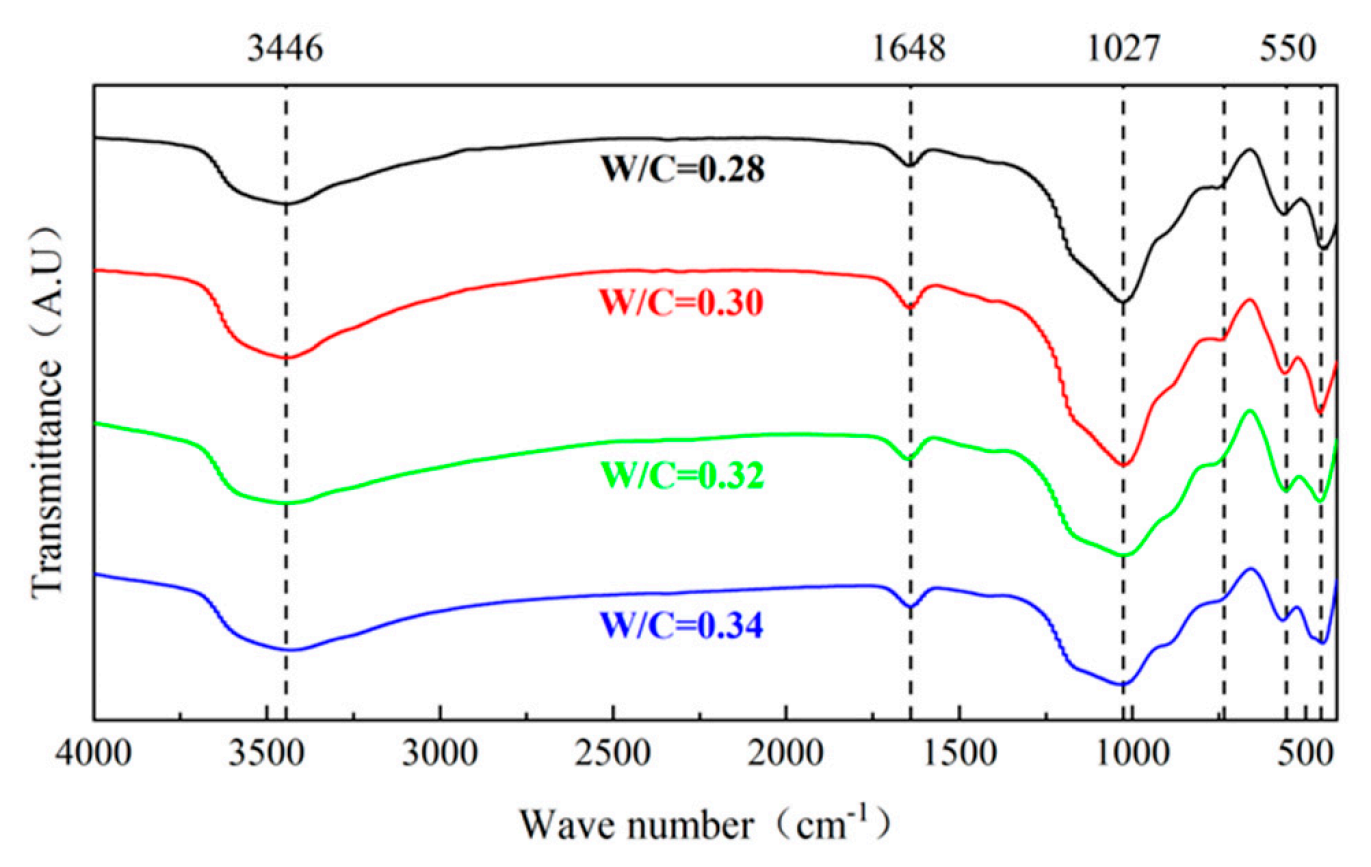
Infrared spectrum of FAG with different W/C.

The FTIR spectra of the FAG with varying W/C ratios, subjected to 90 d of leaching under an applied voltage of 20 V, are presented in Figure 12. As the W/C ratio is elevated, the intensities of both the Si–O–Si and Si–O–Al stretching vibration peaks located at approximately 1027 cm^−1^ and the Si–O–Al bending vibration peak at around 459 cm^−1^ exhibit a trend of initial enhancement followed by subsequent attenuation. The maximum absorption intensities for both peaks are observed at a W/C ratio of 0.30. This indicates that a greater amount of the gel phase is maintained within the leached FAG at this specific mix proportion. A similar trend of initial enhancement followed by attenuation is observed for the O–H bending vibration peak located near 1648 cm^−1^ as the W/C ratio increases; however, the variations in the intensities of these absorption peaks are marginal. This suggests that no significant difference exists in the pore solution alkalinity among the leached FAG specimens with varying W/C ratios, which is consistent with the aforementioned pore solution alkalinity test results.

**Figure 13:**
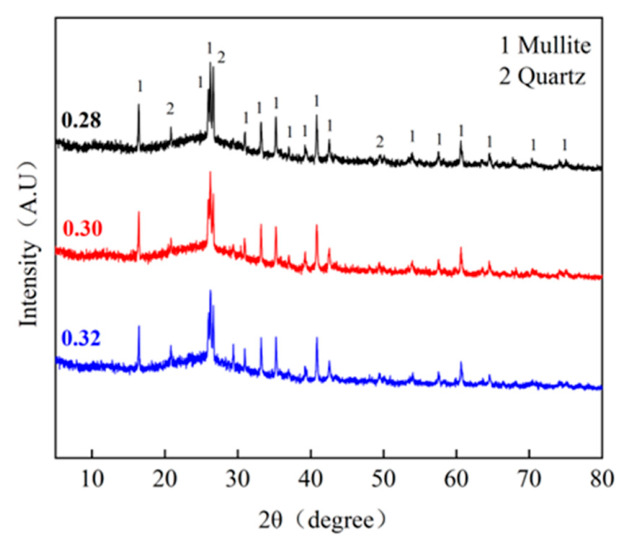
X-ray diffraction pattern of FAG with different W/C.

The XRD patterns of the FAG with varying W/C ratios, subjected to 90 d of leaching under an applied voltage of 20 V, are illustrated in Figure 13. Following the leaching process, quartz and mullite remain the primary crystalline phases within the FAG. No new crystalline phases are generated during the soft water leaching process under the action of stray current due to the variation in the W/C ratio. Furthermore, the intensities of the primary diffraction peaks corresponding to the original crystalline phases are not significantly altered [39].

**Figure 14:**
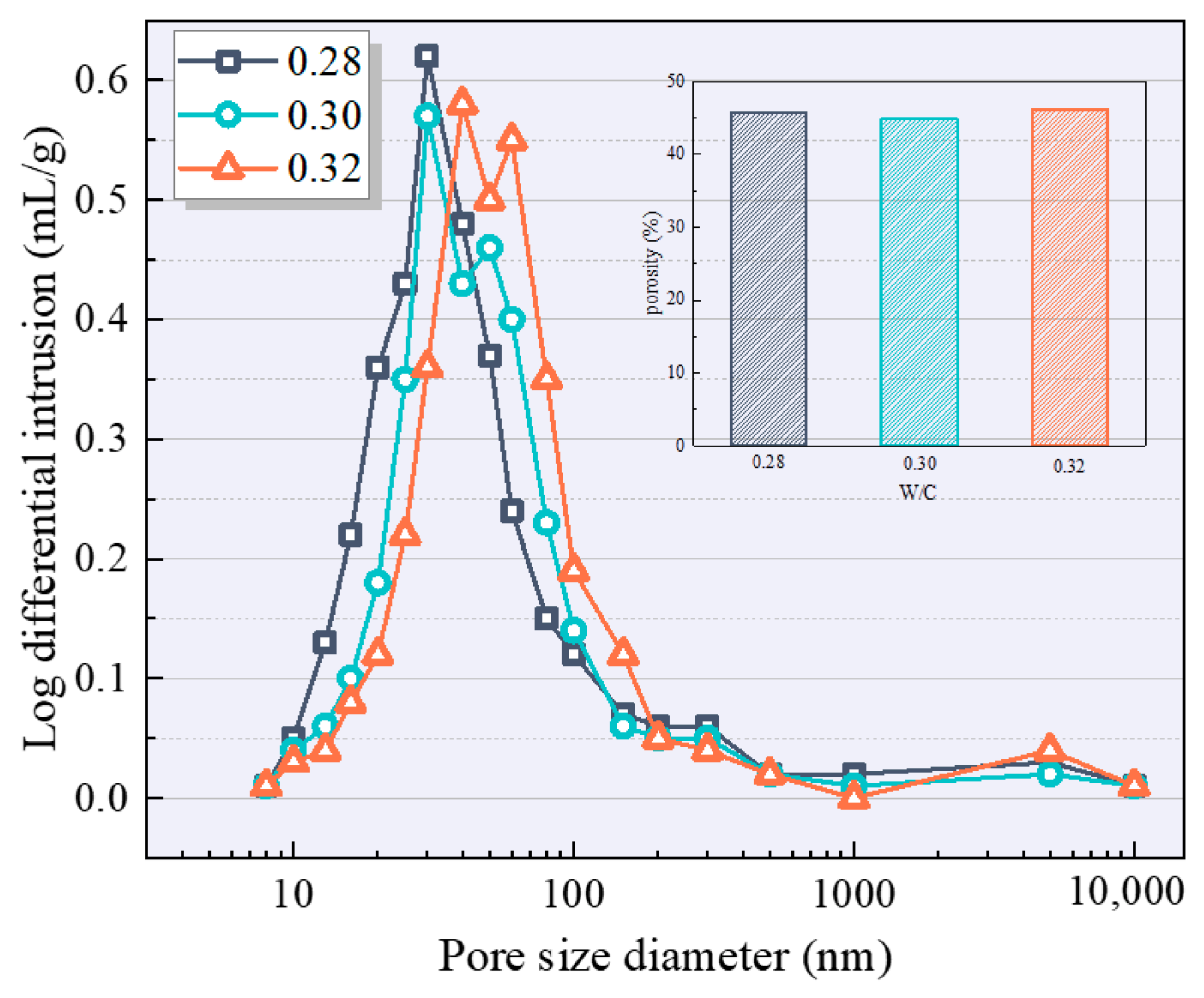
Pore size distribution and porosity of FAG with different W/C.

The pore size distribution and porosity diagrams of the FAG with varying W/C ratios, subjected to 90 d of leaching under an applied voltage of 20 V, are presented in Figure 14. As observed from the figure, when compared to the specimens with a W/C ratio of 0.30, an increase in the quantity of harmful pores with diameters ranging from 105 to 2000 nm is exhibited by the leached FAG with a W/C ratio of 0.28. Similarly, when compared to the group with a W/C ratio of 0.30, an augmentation in the quantity of harmful pores with diameters ranging from 50 to 105 nm is observed in the leached FAG with a W/C ratio of 0.32. At a water-to-binder ratio of 0.30, although the most probable pore diameter of the leached specimens is enlarged to 33.2 nm from the 32.0 nm observed at a ratio of 0.28, the variation between the two remains below 4%. Conversely, when the W/C ratio is increased from 0.30 to 0.32, the most probable pore diameter is significantly enlarged to 41.8 nm, representing a growth rate of 25.9%. The lowest post-leaching porosity of the FAG, recorded at 44.9%, is obtained at a W/C ratio of 0.30. In summary, it is concluded that a superior pore structure is maintained by the FAG following the leaching process when the W/C ratio is optimized at 0.30.

#### 3.2.2. Regulatory Effect of the Sodium Silicate Modulus (Ms)

**Figure 15:**
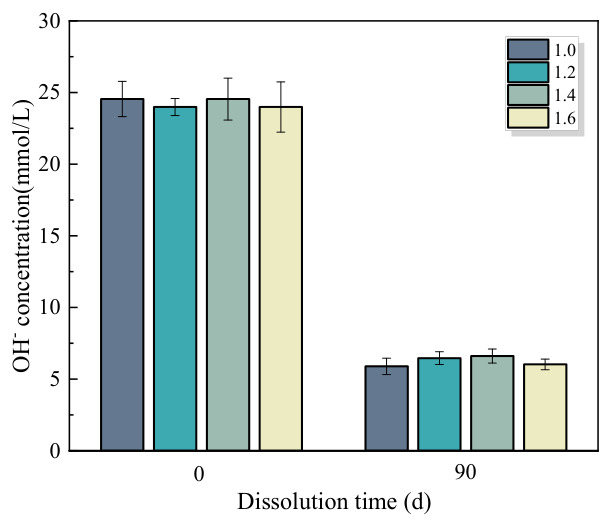
OH^−^ concentration in pore solution of FAG with different Ms before and after corrosion.

**Figure 16:**
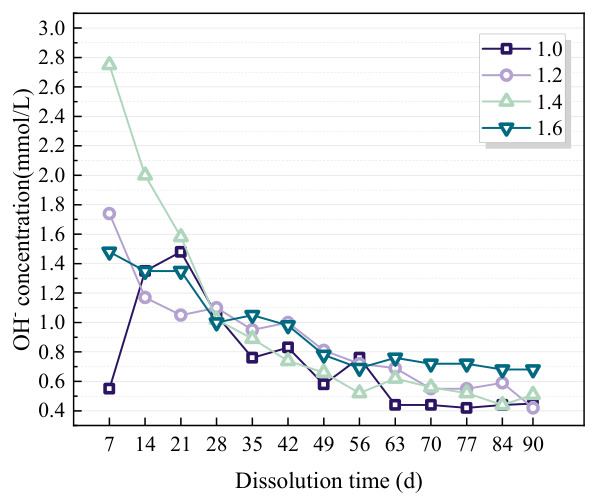
OH^−^ concentration in electrolytic cell solution of FAG with different Ms.

The OH^−^ concentrations in the pore solutions of the FAG with varying Ms before and after leaching under an applied voltage of 20 V are presented in Figure 15. Prior to leaching, a difference of less than 3% is observed between the maximum and minimum OH^−^ concentrations in the pore solutions among the various groups; following leaching, this difference remains below 10%. This indicates that the impact of Ms on the pore solution alkalinity of the FAG prior to leaching is relatively minor [40], whereas its influence on the post-leaching pore solution alkalinity is comparatively more pronounced. The variation in the OH^−^ concentration of the electrolytic cell solution with respect to leaching time for the groups with varying Ms under an applied voltage of 20 V is illustrated in Figure 16. Overall, as the leaching time progresses, a reduction in the amount of OH^−^ leached per unit time is observed across all groups. When the leaching time is less than 28 d, no significant regular pattern is exhibited by the OH^−^ concentration of the electrolytic cell solution. Conversely, when the leaching time exceeds 28 d, the concentration of OH^−^ leached every 7 d is consistently within the range of 0.4 to 1.2 mmol/L. This demonstrates that within this specific leaching period, the amount of OH^−^ leached per unit time is minimally affected by Ms. Across all three designated leaching durations, the minimum cumulative molar amount of leached OH^−^ is consistently recorded for the group with an Ms of 1.0. At a leaching duration of 90 d, the cumulative molar amounts of leached OH^−^ for the groups with Ms of 1.2, 1.4, and 1.6 are 1.19 times, 1.35 times, and 1.28 times that of the group with an Ms of 1.0, respectively. This indicates that although the pore solution alkalinity of the FAG both before and after leaching is not significantly impacted by Ms, the cumulative molar amount of leached OH^−^ is indeed affected. Consequently, a reduction in Ms is conducive to inhibiting the leaching of OH^−^.

**Figure 17:**
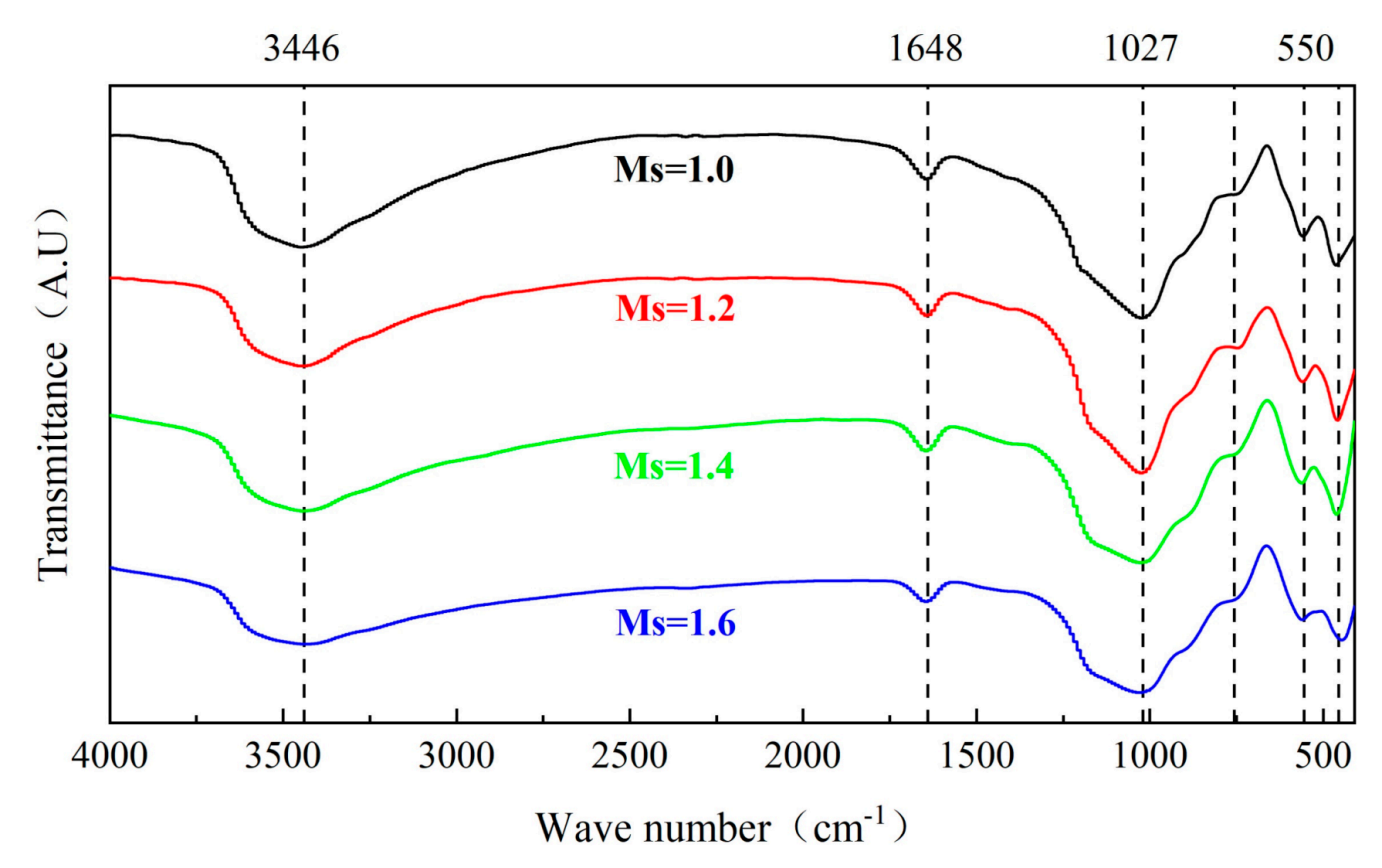
Infrared spectrum of FAG with different Ms.

The FTIR spectra of the FAG with varying Ms, subjected to 90 d of leaching under an applied voltage of 20 V, are presented in Figure 17. As Ms increases, the intensities of both the Si–O–Si and Si–O–Al stretching vibration peaks located at approximately 1027 cm^−1^ and the Si–O–Al bending vibration peak near 459 cm^−1^ exhibit a trend of initial enhancement followed by subsequent attenuation. However, a distinction is observed wherein the maximum absorption intensity for the former is reached at an Ms of 1.2, whereas the peak intensity for the latter is maximized at an Ms of 1.4, although this exhibits only a marginal difference from the peak intensity observed at an Ms of 1.2. This indicates that the maximum amount of the gel phase is maintained within the leached FAG, and the optimal resistance to stray current-induced leaching is achieved when the Ms of the activator is 1.2. Furthermore, the O–H bending vibration peak located near 1648 cm^−1^ fluctuates within a narrow range corresponding to the variation in Ms. This suggests that no significant difference exists in the post-leaching pore solution alkalinity among the FAG specimens with varying Ms.

**Figure 18:**
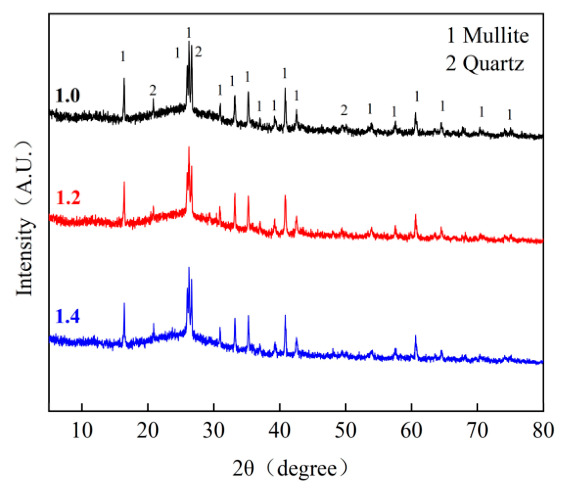
X-ray diffraction pattern of FAG with different Ms.

The XRD patterns of the FAG with varying Ms, subjected to 90 d of leaching under an applied voltage of 20 V, are illustrated in Figure 18. Following the leaching process, quartz and mullite remain the primary crystalline phases within the FAG. No new crystalline phases are generated post-leaching as a result of the variation in Ms [41], nor are the intensities of the primary diffraction peaks corresponding to the original crystalline phases significantly altered.

**Figure 19:**
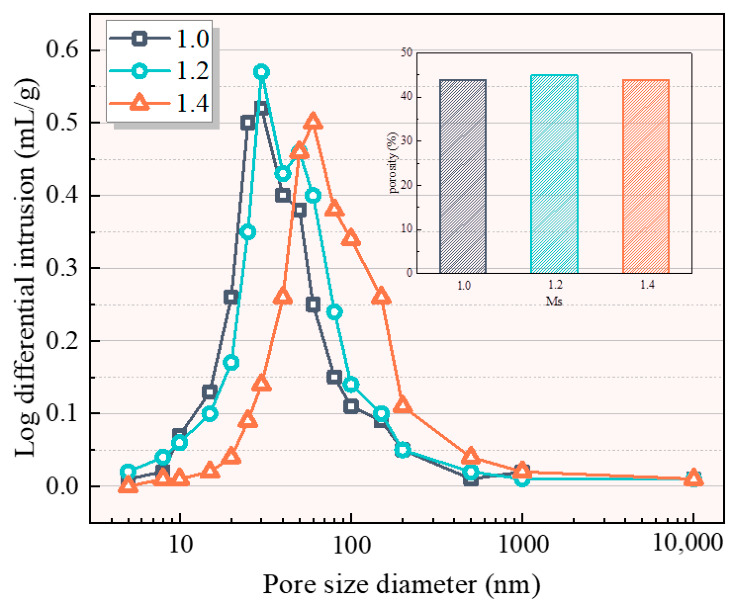
Pore size distribution and porosity of FAG with different Ms.

The pore size distribution and porosity diagrams of the FAG with varying Ms, subjected to 90 d of leaching under an applied voltage of 20 V, are presented in Figure 19. As observed from the figure, an increasing trend in the most probable pore diameter of the leached FAG is exhibited as Ms increases. The minimum most probable pore diameter of the leached FAG, recorded at 29.5 nm, is obtained when Ms is 1.0. When Ms is increased from 1.0 to 1.2, a slight overall shift in the pore size distribution curve toward the larger pore diameter region is observed; however, the two curves essentially overlap in the harmful pore region where the pore diameter is greater than or equal to 110 nm. Concurrently, the most probable pore diameter is enlarged to 33.2 nm, representing a marginal increase of only 12.5%. Conversely, when Ms is increased from 1.2 to 1.4, a substantial shift in the pore size distribution curve toward the larger pore diameter region is exhibited. Furthermore, the quantity of harmful pores with diameters ranging from 80 to 3000 nm is significantly increased, and the most probable pore diameter is enlarged to 58.7 nm, corresponding to a growth of 76.8%. This indicates that the increase in Ms from 1.0 to 1.2 exerts a minimal impact on the post-leaching pore size distribution of the FAG. A relatively small most probable pore diameter is maintained by the FAG following leaching, demonstrating superior resistance to the accelerated leaching induced by stray current. However, when Ms reaches 1.4, both the most probable pore diameter and the quantity of harmful pores within the leached FAG are drastically increased, which is highly detrimental to its mechanical strength. The lowest post-leaching porosity of the FAG, recorded at 43.8%, is observed when Ms is 1.0, whereas the highest porosity of 44.9% is obtained at an Ms of 1.2. A marginal difference of only 1.1% exists between these values, demonstrating that the post-leaching porosity of the FAG is not significantly altered by variations in Ms.

#### 3.2.3. Regulatory Effect of the Alkali Dosage (A/F)

**Figure 20:**
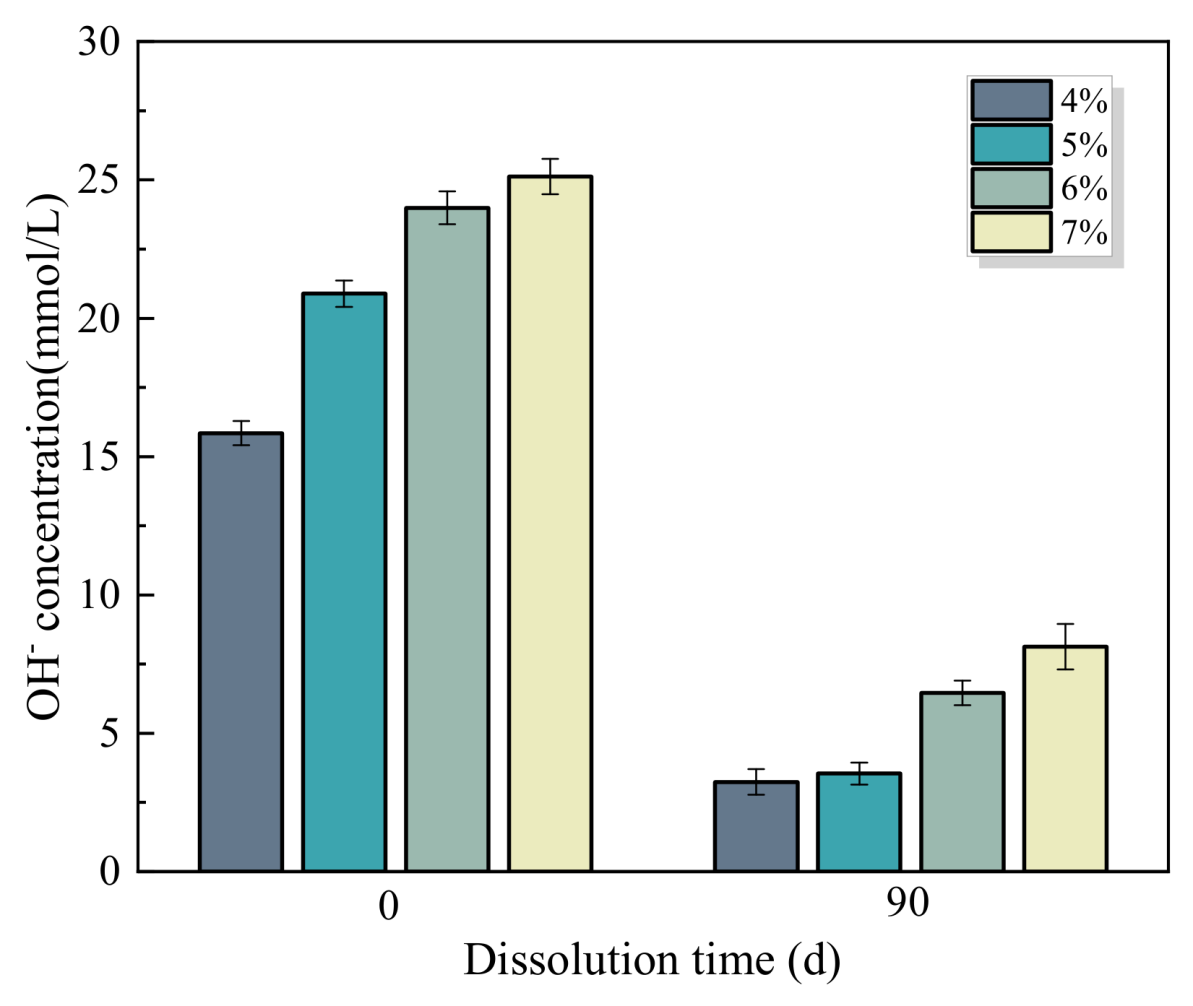
OH^−^ concentration pore solution of FAG with different A/F before and after corrosion.

The OH^−^ concentrations in the pore solutions of the FAG with varying A/F before and after leaching under an applied voltage of 20 V are presented in Figure 20. Prior to leaching, an increase in the pore solution alkalinity of the FAG is observed as A/F increases. The highest pore solution alkalinity is recorded for the FAG with an A/F of 7%, wherein the OH^−^ concentration of the pore solution is 1.58 times that of the group with an A/F of 4%. After 90 d of leaching, the trend of the pore solution alkalinity remains consistent with that observed prior to leaching; at this stage, the OH^−^ concentration of the pore solution for the FAG with an A/F of 7% is 2.51 times that of the group with an A/F of 4%. This indicates that elevating A/F can enhance the pore solution alkalinity of the FAG and is conducive to maintaining a higher pore solution alkalinity post-leaching, which is beneficial for the resistance of the FAG against the coupled leaching of stray current and soft water [42].

**Figure 21:**
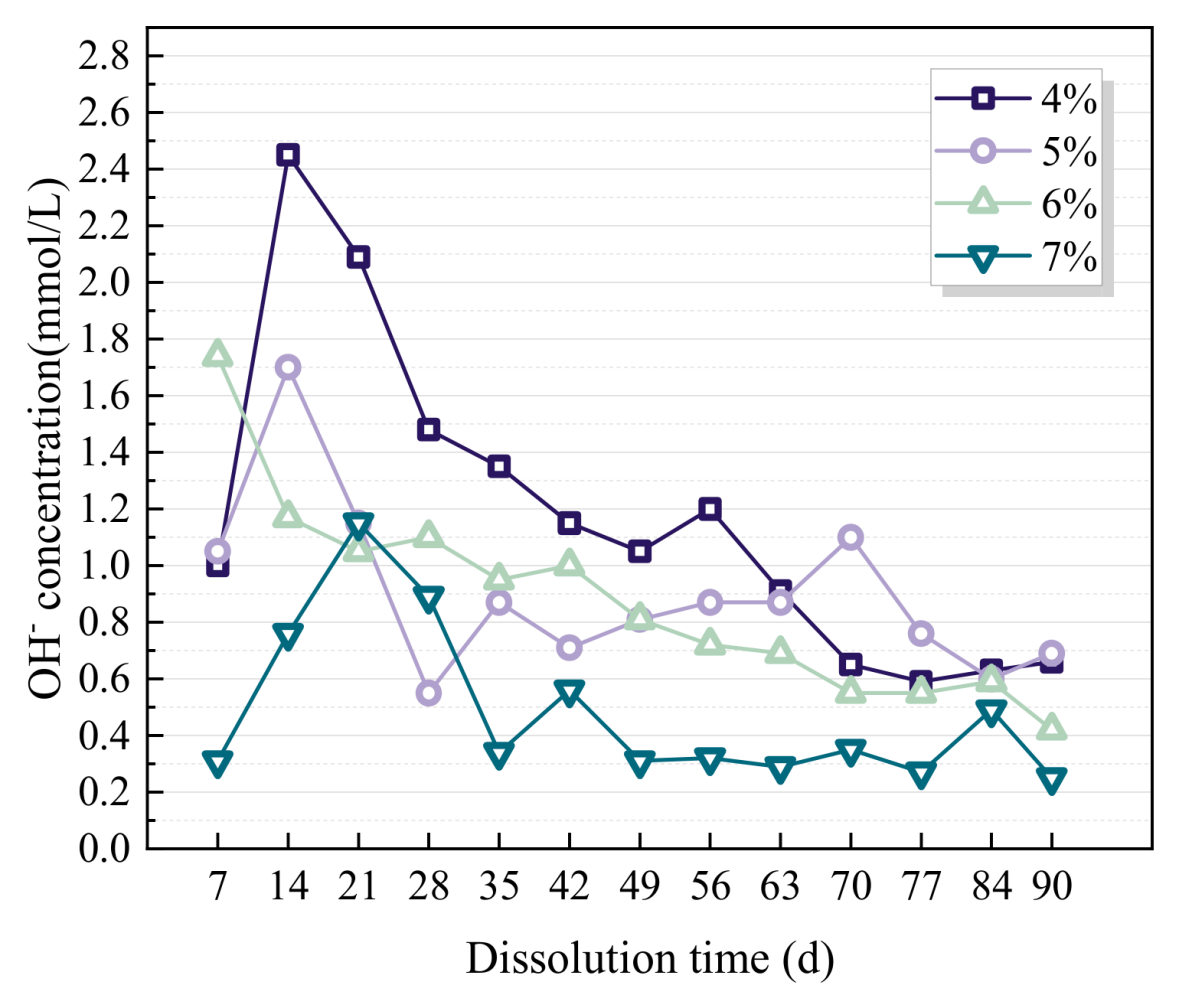
OH^−^ concentration in electrolytic cell solution of FAG with different A/F.

The variation in the OH^−^ concentration of the electrolytic cell solution with respect to leaching time for the groups with varying A/F under an applied voltage of 20 V is illustrated in Figure 21. During the initial stage of leaching, fluctuations are observed in the OH^−^ concentrations of the electrolytic cell solutions across the groups with varying A/F. However, once the leaching time exceeds 21 d, an overall descending trend is exhibited by the OH^−^ concentrations of the electrolytic cell solutions. At 90 d of leaching, a descending trend in the cumulative leached amount of OH^−^ is observed as A/F increases. When A/F is 4%, the cumulative molar amount of leached OH^−^ is 2.42 times that observed at an A/F of 7%. Conversely, at 30 d of leaching, the cumulative molar amount of leached OH^−^ for the group with an A/F of 4% is 2.31 times that of the group with an A/F of 7%, and a relatively high cumulative molar amount of leached OH^−^ is also recorded for the group with an A/F of 6% at this specific duration. Therefore, it is concluded that an inhibitory effect on the leaching of OH^−^ is exerted by elevating A/F, and this inhibitory effect becomes increasingly pronounced as the leaching time progresses.

**Figure 22:**
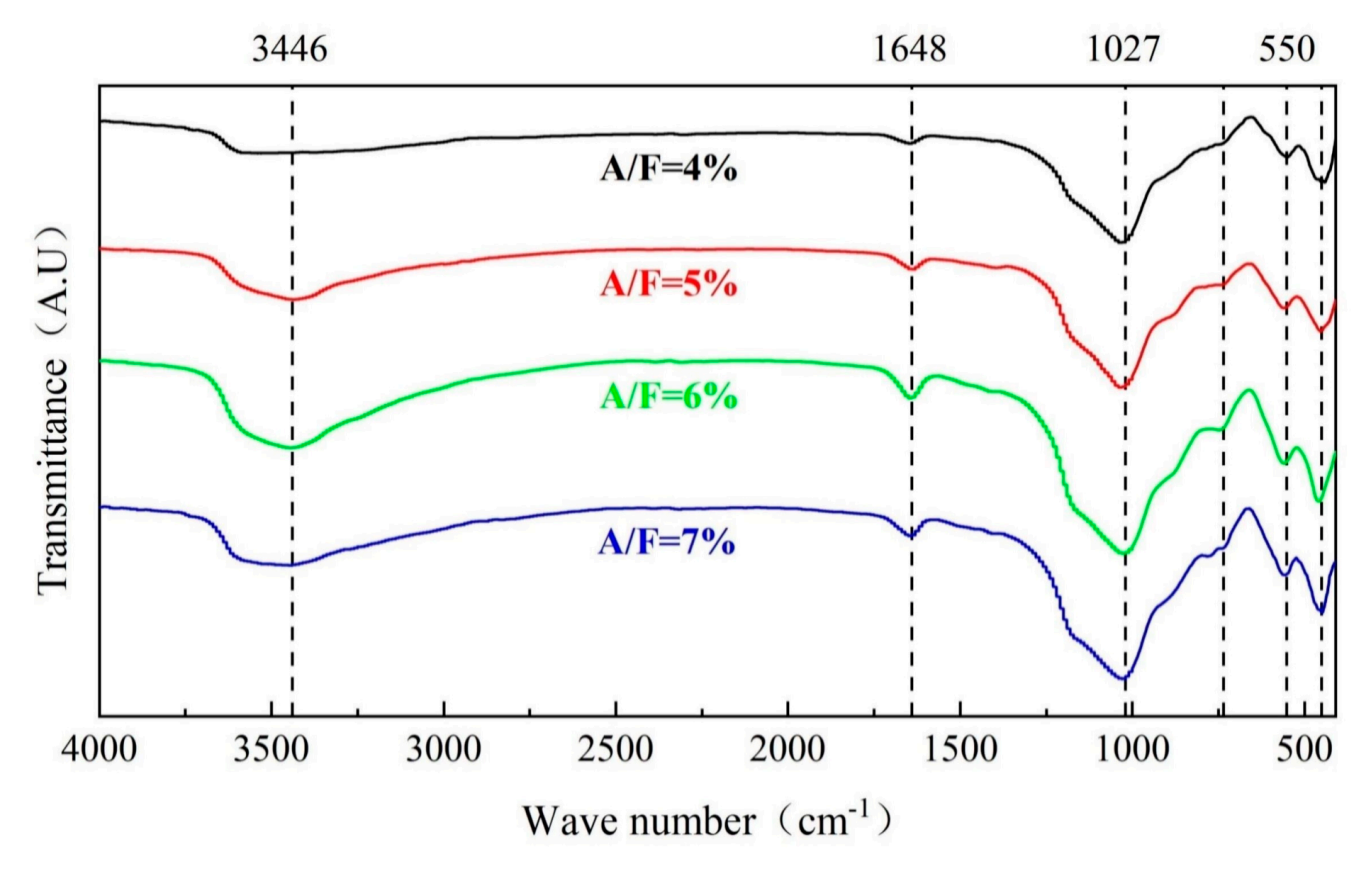
Infrared spectrum of FAG with different A/F.

The FTIR spectra of the FAG with varying A/F, subjected to 90 d of leaching under an applied voltage of 20 V, are presented in Figure 22. As A/F increases, the intensities of the Si–O–Si and Si–O–Al stretching vibration peaks located at approximately 1027 cm^−1^ exhibit a trend of initial enhancement followed by subsequent attenuation, with the maximum absorption peak observed at an A/F of 6%. An increasing trend is exhibited by the intensity of the Si–O–Al bending vibration peak near 459 cm^−1^ as A/F is elevated; however, a marginal difference is observed between the intensities of the absorption peaks at A/F of 6% and 7%. This indicates that the maximum amount of the gel phase is maintained within the leached FAG, and the optimal resistance to stray current-induced leaching is achieved when A/F is 6%. Furthermore, the O–H bending vibration peak located near 1648 cm^−1^ exhibits a trend of initial enhancement followed by attenuation as A/F increases. The intensities of this absorption peak at A/F of 6% and 7% are significantly higher than those observed at A/F of 4% and 5%, demonstrating that a relatively high pore solution alkalinity and a greater quantity of the gel phase can be maintained post-leaching when the A/F of the FAG is 6% or higher.

**Figure 23:**
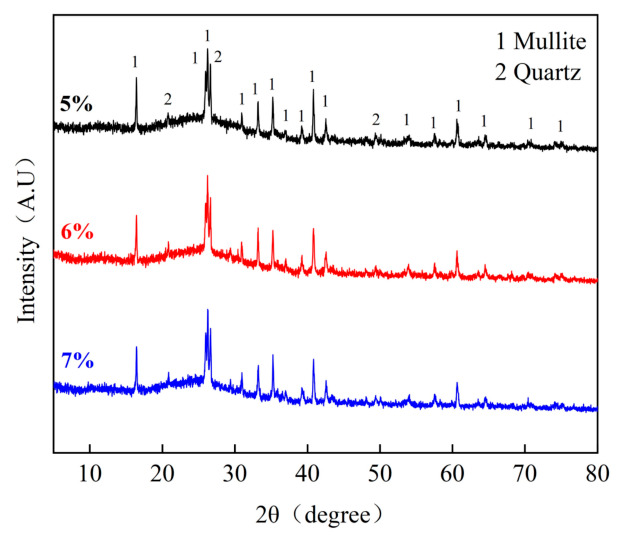
X-ray diffraction pattern of FAG with different A/F.

The XRD patterns of the FAG with varying A/F, subjected to 90 d of leaching under an applied voltage of 20 V, are illustrated in Figure 23. Following the leaching process, quartz and mullite remain the primary crystalline phases within the FAG. No new crystalline phases are generated post-leaching as a result of the variation in A/F, nor are the intensities of the primary diffraction peaks corresponding to the original crystalline phases significantly altered.

**Figure 24:**
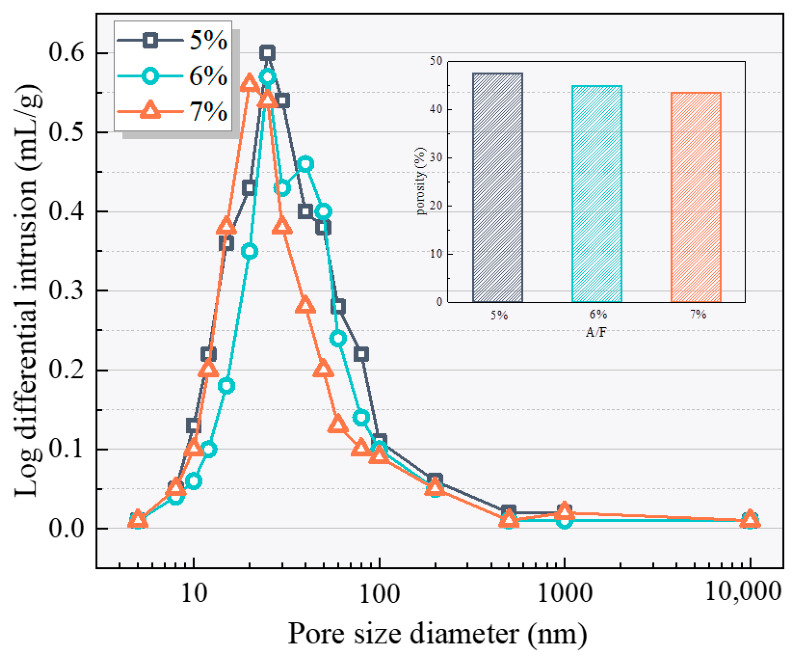
Pore size distribution and porosity of FAG with different A/F.

The pore size distribution and porosity diagrams of the FAG with varying A/F, subjected to 90 d of leaching under an applied voltage of 20 V, are presented in Figure 24. As observed from the figure, the pore size distribution curves for the FAG with A/F of 6% and 7% essentially overlap within the harmful pore range where the pore diameter exceeds 150 nm. This indicates that the quantity of harmful pores within this range is virtually identical for these two alkali dosages. Conversely, when the A/F is reduced to 5%, a significant increase in the quantity of harmful pores with diameters greater than 150 nm is observed. With the elevation of A/F, a decreasing trend in the most probable pore diameter of the leached FAG is exhibited. Specifically, as the A/F increases from 5% to 7%, the most probable pore diameter is reduced from 33.4 nm to 28.3 nm. The lowest post-leaching porosity of the FAG, recorded at 43.6%, is observed at an A/F of 7%, whereas the highest porosity of 47.6% is obtained at an A/F of 5%. Overall, a descending trend in porosity is exhibited as the A/F increases. This indicates that, within the assumptions and comparative resolution of the MIP method, FAG with a higher A/F retained a lower mercury-accessible porosity and a finer apparent pore-throat distribution after leaching.

Fly ash is an intrinsically variable industrial by-product. Differences in bulk Si/Al ratio, Ca content, amorphous-phase fraction, quartz and mullite contents, loss on ignition, particle-size distribution, and surface area may alter precursor dissolution, gel formation, initial pore structure, and ion-transport resistance. Consequently, the absolute leaching rate and the mixture proportions identified as favorable in this study should not be directly generalized to all fly-ash sources.

The proposed deterioration pathway is expected to be qualitatively relevant to low-calcium, N–A–S–H-dominated fly-ash geopolymers, but its quantitative parameters require source-specific calibration. Future studies should compare fly ashes from multiple power plants and production batches and should include quantitative amorphous-phase analysis, particle-size distribution, specific surface area, and reactivity tests.

## 4. Conclusions

In this study, the alkalinity of the pore solution and the OH^−^ concentration of the electrolytic cell solution were employed as evaluation indicators. Combined with microstructural characterization techniques, including XRD, SEM, MIP, and FTIR, the mechanisms underlying the influence of the W/C ratio, Ms, and A/F on the leaching behavior of FAG were elucidated. The primary conclusions are as follows:

(1) Leaching degradation mechanism: The leaching of OH^−^ from the FAG pore solution is accelerated by the stray current, leading to a precipitous decline in alkalinity, which subsequently destroys the N-A-S-H gel network responsible for the binding strength. The decomposition of the gel phase directly results in increased porosity, an enlarged most probable pore diameter, and a proliferation of harmful pores (diameter ≥ 50 nm), which constitute the fundamental microstructural causes of macroscopic strength deterioration.
(2) Phase stability and kinetic characteristics: The crystalline phases within the FAG system are not destroyed by the stray current; the original crystals remain stable during the leaching process, and no new crystalline phases are generated. Furthermore, the coupled leaching effect of stray current and soft water exhibits a time-dependent attenuation. Specifically, the coupled destructive effect weakens with prolonged leaching time, and the leaching rate of OH^−^ exhibits a linear decreasing trend.
(3) Superior erosion resistance at lower W/C ratios: The cumulative molar amount of leached OH^−^ can be significantly reduced by lowering the W/C ratio, thereby enhancing the leaching resistance of the FAG. Comprehensive evaluation indicates that at a W/C ratio of 0.30, the FAG not only maintains a higher pore solution alkalinity post-leaching but also retains a greater quantity of the gel phase and a superior pore structure, yielding the optimal overall leaching resistance.
(4) Structural stability favored by lower Ms: Reducing Ms facilitates the inhibition of OH^−^ leaching and optimizes the micro-morphology after leaching. The minimum leached amount and the densest pore structure (characterized by the lowest porosity and fewer harmful pores) are achieved in the FAG with an Ms of 1.0, whereas an Ms of 1.2 is more conducive to maintaining the pore solution alkalinity and gel phase content. Overall, a lower Ms (1.0–1.2) is more beneficial for resisting coupled leaching.
(5) Positive inhibitory effect of increasing A/F: The resistance of the FAG to stray current-induced leaching is effectively enhanced by increasing the A/F. A higher A/F not only improves the initial pore solution alkalinity of the structure but also strongly inhibits the loss of OH^−^ during the leaching process. Moreover, this inhibitory effect becomes increasingly significant over time, allowing the system to maintain a high-alkalinity environment even after prolonged leaching.

Overall, the deterioration of low-calcium FAG under coupled stray current and soft water is mainly attributed to pore-solution alkalinity loss, subsequent N–A–S–H degradation, and pore-network deterioration, while quartz and mullite remain comparatively stable. Within the investigated ranges, W/C ≈ 0.30, Ms = 1.0–1.2, and A/F = 6–7% provided the most favorable resistance to coupled leaching. However, these ranges are specific to the fly ash, activator, constant 20 V DC field, deionized-water renewal protocol, and 90 d exposure adopted in this study, and should not be regarded as universally optimal. Further validation under realistic groundwater chemistry, fluctuating stray-current conditions, and multiple fly-ash sources is required before field service-life prediction.

## Figures and Tables

**Table 1:** The physical properties and main chemical components of fly ash.

Physical Properties/Chemical Components	Fly Ash
Density (g/cm^3^)	2.3
Moisture content (%)	0.7
Chemical composition (%)	
CaO	3.71
SiO_2_	53.86
Al_2_O_3_	29.45
ZrO_2_	0.09
Fe_2_O_3_	5.46
SrO	0.08
MgO	0.62
TiO_2_	2.04
ZnO	0.06
P_2_O_5_	0.54
SO_3_	1.84
BaO	0.23
K_2_O	1.53
Na_2_O	0.44
CuO	0.02
MnO	0.02

**Table 2:** Main technical specifications of water glass.

Parameter	Fly Ash
Modulus	3.3
Baumé degree (°Bé)	39.9
Density (g/mL)	1.380
Transparency (%)	92.6
Na_2_O (%)	8.82
SiO_2_ (%)	28.26
Fe (%)	0.004

**Table 3:** NaOH dosage of water glass with different modulus.

Modulus of Water Glass	1.0	1.2	1.4	1.6	1.8	2.0
NaOH dosage	26.30	20.02	15.53	12.17	9.55	7.46

**Table 4:** FAG Mix Proportion Design Table.

Group	Code	W/C	Ms	A/F (%)	Water Glass (g)	NaOH (g)	Water (g)
W/C variable	W1	0.28	1.2	6	246.6	49.4	124.9
	reference	0.3	1.2	6	246.6	49.4	144.9
	W2	0.32	1.2	6	246.6	49.4	164.9
	W3	0.34	1.2	6	246.6	49.4	184.9
Ms variable	M1	0.3	1	6	205.5	54	170.7
	reference	0.3	1.2	6	246.6	49.4	144.9
	M2	0.3	1.4	6	287.7	44.7	119
	M3	0.3	1.6	6	328.7	40	93.2
A/F variable	A1	0.3	1.2	4	164.4	32.9	196.6
	A2	0.3	1.2	5	205.5	41.1	170.7
	reference	0.3	1.2	6	246.6	49.4	144.9
	A3	0.3	1.2	7	287.7	57.6	119

## Data Availability

The original contributions presented in this study are included in the article. Further inquiries can be directed to the corresponding author.

## References

[1] Wang Y., Li M., Zhou J., Zheng H. (2022). Sudden passenger flow characteristics and congestion control based on intelligent urban rail transit network. Neural Comput. Appl..

[2] Wu P., Zhang Z., Peng X., Wang R. (2024). Deep learning solutions for smart city challenges in urban development. Sci. Rep..

[3] Bi S., Hu J., Shao L., Feng T., Appolloni A. (2024). Can public transportation development improve urban air quality? Evidence from China. Urban Clim..

[4] Wang C., Li W., Wang Y., Xu S., Fan M. (2018). Stray current distributing model in the subway system: A review and outlook. Int. J. Electrochem. Sci..

[5] Chen Z., Koleva D., van Breugel K. (2017). A review on stray current-induced steel corrosion in infrastructure. Corros. Rev..

[6] Wang C., Qin G. (2024). Corrosion of underground infrastructures under metro-induced stray current: A review. Corros. Commun..

[7] Gui P., Jin H., Yu S. (2025). Study on corrosion resistance of the steel spring floating slab using rubber concrete under stray current. Constr. Build. Mater..

[8] Zhang B. (2024). Durability of low-carbon geopolymer concrete: A critical review. Sustain. Mater. Technol..

[9] Manzoor T., Bhat J.A., Shah A.H. (2024). Performance of geopolymer concrete at elevated temperature—A critical review. Constr. Build. Mater..

[10] Liu Y., Hu X., Du Y., Nematollahi B., Shi C. (2024). A review on high-temperature resistance of geopolymer concrete. J. Build. Eng..

[11] Xie J., Zhao J., Wang J., Wang C., Huang P., Fang C. (2019). Sulfate resistance of recycled aggregate concrete with GGBS and fly ash-based geopolymer. Materials.

[12] Guo L., Wu Y., Xu F., Song X., Ye J., Duan P., Zhang Z. (2020). Sulfate resistance of hybrid fiber reinforced metakaolin geopolymer composites. Compos. Part B Eng..

[13] Shahedan N.F., Hadibarata T., Abdullah M.M.A.B., Jusoh M.N.H., Abd Rahim S.Z., Isia I., Bras A.A., Bouaissi A., Juwono F.H. (2024). Potential of fly ash geopolymer concrete as repairing and retrofitting solutions for marine infrastructure: A review. Case Stud. Constr. Mater..

[14] Chenna H.N.P., Chouksey S.K., Turkane S.D. (2025). Performance evaluation and optimization of landfill waste-fly ash geopolymer for road subgrade applications. Constr. Build. Mater..

[15] Zhao P., Xu G., Wang Q., Zeng Z. (2024). Corrosion behavior of steel bars in simulated concrete pore solution under the coupling action of chloride salt and DC stray current. Constr. Build. Mater..

[16] Chen M., Kang X., Chen Y., Chen R. (2025). Unveiling the synergistic effects of stray current and high hydraulic pressure on chloride transport in ultra-high-performance concrete. Cem. Concr. Compos..

[17] Chen M.Y., Chen R.P., Chen Y.Q., Ma X.Y., Kang X. (2023). Stray current induced chloride ion transport and corrosion characteristics of cracked ultra-high performance concrete. Constr. Build. Mater..

[18] Saito H., Tajima T., Fujiwara A., Tsuji Y. (1997). Application of electrochemical acceleration test method to changes in cementitious barrier performances by leaching degradation. MRS Online Proc. Libr. (OPL).

[19] Bertolini L., Carsana M., Pedeferri P. (2007). Corrosion behaviour of steel in concrete in the presence of stray current. Corros. Sci..

[20] Luo Y., Wang C., Luo C., Huang Q., Wang S., Peng X. (2016). Effect of electrical field on TSA failure of cement-based materials. Cem. Concr. Res..

[21] Huang Q., Wang C., Yang C., Zhou L., Yin J. (2015). Accelerated sulfate attack on mortars using electrical pulse. Constr. Build. Mater..

[22] Huang Q., Wang C., Zeng Q., Yang C., Luo C., Yang K. (2016). Deterioration of mortars exposed to sulfate attack under electrical field. Constr. Build. Mater..

[23] Huang Q., Wang C., Luo C., Yang C., Luo Y., Xie H. (2017). Effect of mineral admixtures on sulfate resistance of mortars under electrical field. Adv. Cem. Res..

[24] Wang S., Cao J., Gong F., Peng Y., Wang Z., Zhao Y., Zeng B. (2024). Insights on the multiple ions distribution in concrete under stray current: From experiments to multi-field simulation. J. Build. Eng..

[25] Cheng X., Liu X., Xiang E., Chen M., Ma C. (2025). Effects of Stray Current on Chloride Ingress in Underground Reinforced Concrete Structures. Buildings.

[26] Chen Y., Chen M., Chen R., Kang X. (2023). Deterioration mechanism of chloride attack on reinforced concrete under stray current and high hydraulic pressure coexistence environment. Mater. Struct..

[27] (2022). Sodium Silicate for Industrial Use.

[28] Sun J., Chen Z. (2019). Effect of silicate modulus of water glass on the hydration of alkali-activated converter steel slag. J. Therm. Anal. Calorim..

[29] Tang R., Liu F., Wang B., Wang X., Hua C., Yuan X. (2025). Mechanical Behavior of Fly-Ash Geopolymer Under Stray-Current and Soft-Water Coupling. Buildings.

[30] Hua C., Tang R., Lu X. (2024). Radon emission characteristics and pore structure evolution of self-compacting concrete with silica fume-molybdenum tailings under different curing environments. J. Build. Eng..

[31] Chen Y., Al-Neshawy F., Punkki J. (2021). Investigation on the effect of entrained air on pore structure in hardened concrete using MIP. Constr. Build. Mater..

[32] Ma H. (2014). Mercury intrusion porosimetry in concrete technology: Tips in measurement, pore structure parameter acquisition and application. J. Porous Mater..

[33] Nath S.K., Kumar S. (2017). Reaction kinetics, microstructure and strength behavior of alkali activated silico-manganese (SiMn) slag–Fly ash blends. Constr. Build. Mater..

[34] Fernández-Jiménez A., Palomo A., Criado M. (2005). Microstructure development of alkali-activated fly ash cement: A descriptive model. Cem. Concr. Res..

[35] Yang H., Wang X., Wang E., Song Y., Chen D., Zhang Y., Liu W. (2024). Macro-micro crack and damage evolution characteristics of concrete: After the action of acidic drying-saturation cycle. Constr. Build. Mater..

[36] Ren J., Luo X., Bai R., Pan C., Zhang J. (2022). Pore characteristics of different phase in nano-modified concrete and their influences on the compressive strength. J. Build. Eng..

[37] Shi X., Feng Y., Zhang Y., Su Y. (2023). A comprehensive investigation on sulphate resistance of geopolymer recycled concrete: Macro and micro properties. Constr. Build. Mater..

[38] Natkunarajah K., Masilamani K., Maheswaran S., Lothenbach B., Amarasinghe D.A.S., Attygalle D. (2022). Analysis of the trend of pH changes of concrete pore solution during the hydration by various analytical methods. Cem. Concr. Res..

[39] Li Y., Xu W., Li H., Lai J., Qiang S., Luo T. (2022). Multi-ion erosion experiment and corrosion mechanism verification of steel fiber–reinforced concrete under stray current. J. Mater. Civ. Eng..

[40] Tan Y., He Y., Cui X., Liu L. (2024). The influence of different water glass moduli on the chemical corrosion resistance of alkali-activated porous concrete. Constr. Build. Mater..

[41] Liu J., Guo L., Cheng L., Xi Y., Chen D. (2024). Effect of alkali dosage and silicate modulus on the deterioration of alkali-activated concrete properties subjected to sodium chloride attack and freeze thaw cycles. Constr. Build. Mater..

[42] Lei J., Kumar D., Yang E.H. (2025). Effect of pore solution alkalinity on alkali–silica reaction (ASR) in metakaolin-based geopolymer. Struct. Concr..

